# Machine learning enabled design features of antimicrobial peptides selectively targeting peri-implant disease progression

**DOI:** 10.3389/fdmed.2024.1372534

**Published:** 2024-04-05

**Authors:** Kyle Boone, Natalia Tjokro, Kalea N. Chu, Casey Chen, Malcolm L. Snead, Candan Tamerler

**Affiliations:** 1 Institute for Bioengineering Research, University of Kansas, Lawrence, KS, United States; 2 Department of Mechanical Engineering, University of Kansas, Lawrence, KS, United States; 3 Center for Craniofacial Molecular Biology, Herman Ostrow School of Dentistry of USC, University of Southern California, Los Angeles, CA, United States; 4 Bioengineering Program, University of Kansas, Lawrence, KS, United States

**Keywords:** machine learning, antimicrobial peptides, peri-implantitis, targeting, bacterial resistance, oral health, rational design

## Abstract

Peri-implantitis is a complex infectious disease that manifests as progressive loss of alveolar bone around the dental implants and hyper-inflammation associated with microbial dysbiosis. Using antibiotics in treating peri-implantitis is controversial because of antibiotic resistance threats, the non-selective suppression of pathogens and commensals within the microbial community, and potentially serious systemic sequelae. Therefore, conventional treatment for peri-implantitis comprises mechanical debridement by nonsurgical or surgical approaches with adjunct local microbicidal agents. Consequently, current treatment options may not prevent relapses, as the pathogens either remain unaffected or quickly re-emerge after treatment. Successful mitigation of disease progression in peri-implantitis requires a specific mode of treatment capable of targeting keystone pathogens and restoring bacterial community balance toward commensal species. Antimicrobial peptides (AMPs) hold promise as alternative therapeutics through their bacterial specificity and targeted inhibitory activity. However, peptide sequence space exhibits complex relationships such as sparse vector encoding of sequences, including combinatorial and discrete functions describing peptide antimicrobial activity. In this paper, we generated a transparent Machine Learning (ML) model that identifies sequence-function relationships based on rough set theory using simple summaries of the hydropathic features of AMPs. Comparing the hydropathic features of peptides according to their differential activity for different classes of bacteria empowered predictability of antimicrobial targeting. Enriching the sequence diversity by a genetic algorithm, we generated numerous candidate AMPs designed for selectively targeting pathogens and predicted their activity using classifying rough sets. Empirical growth inhibition data is iteratively fed back into our ML training to generate new peptides, resulting in increasingly more rigorous rules for which peptides match targeted inhibition levels for specific bacterial strains. The subsequent top scoring candidates were empirically tested for their inhibition against keystone and accessory peri-implantitis pathogens as well as an oral commensal bacterium. A novel peptide, VL-13, was confirmed to be selectively active against a keystone pathogen. Considering the continually increasing number of oral implants placed each year and the complexity of the disease progression, prevalence of peri-implant diseases continues to rise. Our approach offers transparent ML-enabled paths towards developing antimicrobial peptide-based therapies targeting the changes in the microbial communities that can beneficially impact disease progression.

## 1 Introduction

Despite high success rates for dental implants, their bacterial plaque-associated inflammatory lesions, known as peri-implant diseases, still occur (1, 2). These lesions continue to degrade the stability of peri-implant soft and hard tissues, which can result in loss of the implant. While peri-implant mucositis is a reversible inflammatory condition, peri-implantitis is an irreversible pathological condition leading to loss of supporting alveolar bone (2). The reported prevalence of peri-implant mucositis and periimplantitis shows a substantial increase over time following implant placement. Meta-analysis for patient-based peri-implant mucositis and peri-implantitis was reported as 46.83% and 19.83% by Lee *et al*. (3) In a separate study, meta-analyses estimated the peri-implant mucositis and peri-implantitis as 43% and 22%, respectively. Peri-implantitis is also reported to be in the range of 11%–47% among dental implants 10 years after their placement (4, 5). These numbers further increase in periodontally compromised patients (1, 6, 7).

Current treatments for peri-implantitis and periodontitis include mechanical debridement, disinfection of exposed implant surfaces, and antibiotic or antiseptic prescription to suppress the associated bacteria (5). The use of adjunctive antibiotics for treating peri-implantitis or periodontitis is debated mainly due to the concern of microbial antibiotic resistance, the non-selective suppression of both pathogenic and commensal species, and the adverse systemic reactions. Notably, these conventional treatment modalities may not prevent relapses, as the pathogens may either remain unaffected or quickly re-emerge after treatment.

The poor efficacy of antibiotic treatment in peri-implantitis may be explained by their non-specific suppression of dysbiotic biofilms. The adaptability and resiliency of pathogenic bacteria in biofilms is well documented (8). The unique structure and inter-species relationships within a biofilm enhance the individual strengths of the bacteria present, creating an unbalanced community organized to promote communal success at the expense of the host (9). Notably, keystone pathogens play an outsized role in shaping the community structure. Therefore, targeting keystone pathogens may be the most effective approach to reverse microbial dysbiosis and return to health-compatible eubiosis.

In peri-implantitis, *Porphyromonas gingivalis* (*P. gingivalis*) is widely acknowledged as a keystone pathogen (10). *P. gingivalis* is associated with increased levels of inflammation and subsequent alveolar bone loss (11). Once *P. gingivalis* has initiated biofilm growth, other pathogens are free to flourish and further contribute to the dysbiotic community (11, 12). The interdependent-relationships among pathogens in a biofilm are a defining factor in their treatment difficulty. In peri-implantitis, this is evident by the coexistence of *Aggregatibacter actinomycetemcomitans*, another keystone pathogen associated with aggressive periodontitis, and *Streptococcus gordonii*, a commensal and accessory pathogen (13, 14). Microbial communities exhibiting both *P. gingivalis* and *S. gordonii* are linked to more severe cases of peri-implantitis, resulting in increased infection and bone loss as compared to others (15–17). Undoubtedly, in peri-implantitic biofilms, pathogens grow synergistically to promote each other’s survival. Keystone pathogens, such as *P. gingivalis* and *A. actinomycetemcomitans,* play a pivotal role in shifting the oral microbiome to induce the host into a disease-oriented state. The presence of these pathogens is widely associated with intensified inflammation levels, prolonged infection, and enhanced alveolar bone loss in patients (18, 19). Successful mitigation of disease progression in peri-implantitis requires a specific mode of treatment capable of targeting keystone pathogens and restoring bacterial community balance toward commensal species. Broad spectrum approaches have difficulty in preventing oral dysbiosis (11, 18, 20).

Antimicrobial peptides (AMPs) have been receiving increasing attention as promising therapeutic candidates since their use lead to no or low antibiotic resistance (21, 22). Moreover, AMPs with a short sequence domain offer straight-forward manufacturing, and relatively low cost production (23). Within AMPs, antibacterial peptides account for the largest proportion of peptides with inhibitory activities ranging from broad- to specific-species (21). Complexity in structure-function relationships in AMPs is increasing with the increased number of peptide sequences that are isolated from a wide range of organisms, or designed using computational search methods, or developed as peptide-mimics as potential candidates (24–27). Despite such progress, and the promise of AMPs as alternative treatments to antibiotics and antiseptics, still only a handful of AMPs have been applied to oral-craniofacial applications (28–33). We and other groups investigated AMPs that may serve to reduce biofilm load and/or to target emergent keystone pathogens on dental implants, and mitigation of bacterial-induced peri-implantitis has been demonstrated by rationally designed chimeric AMPs and peptides (30, 34–40).

As a result of growing interest in AMPs, large databases on their sequence and known functions are now readily available (41–46). With the increasing number of AMPs discovered at the lab bench and via computational methods, determining the boundaries of similar AMPs and identifying their bacteria-specific function remains challenging. Machine learning models offer unique tools to find AMPs with targeted function (42). Through bioinformatics similarity tools, ML models are effective in identifying possible antimicrobial peptides among the large number of nucleic acids sequences (41). Recurrent neural networks (RNNs), long-short term models (LSTMs) and other deep learning methods have demonstrated success in peptide related-prediction models and these methods are now being used in constructive model approaches to design AMPs (47–51). However, when these methods are applied in generative approaches, they severely lack training adaptability. This is mainly due to their requirement for a relatively large number of training sets needed to learn specific paths in high-dimensional decision space. This makes them at risk for re-enforcing errors when the models incorrectly classify cases by correlated features. Customizing decisions for the sampled distribution may optimize prediction performance, but this makes the ability of the model to adjust to the trends in unbiased sampling data extremely difficult to achieve. Gradient-descent or back-propagating methods can help to recognize the contribution from previously under-represented subgroups that are negatively impacting the models’ applicability and overall efficacy. However, since retraining the whole model comes with a large computational cost, alternative methods are sought to address the bias. Still, no strategies are currently known which resolve this issue.

In our prior art, we pioneered the use of rough set theory for the classification of peptide sequences and demonstrated how to achieve training adaptability by bypassing neural networks in the context of AMP identification (52). In this approach, different descriptors in accordance with antibacterial activity are analyzed by rough set theory (RST) which is used as a heuristic method to discover the rules distinguishing different outcomes. By combining the rough set theory approach with the algorithm of Modified Learning from Examples Module, Version 2 (MLEM2) and the Interesting Rule Induction Module (IRIM) algorithm, we achieved high-specificity performance. Our method provides a transparent selection approach to define explicit boundaries that distinguish between classes of AMPs by their activity. The model can adapt by using the explicit decision components and the related rules that are introduced by new hypotheses and labelled data. Non-linear categories distinguishing between active and inactive peptides reduce training time of the model, while preserving the structure of the explicit model choices. This improves training flexibility, as well as avoids the cost barrier associated with re-training or the creation of new models. This method also guards against irrational decision relationships by maintaining transparency for each decision step throughout the decision process. In a separate study, we combined the RST based ML approach (CLN-MLEM2) with a codon-based genetic algorithm (CB-GA) and increased the variations of peptide sequences generated by RST ML search (53). Using the CB-GA combined ML approach, we identified an AMP sequence effective against Staphylococcus epidermidis. The training false discovery rate, i.e., probability of false positives, was approximately 5% (53).

In this study, we developed a transparent ML model, combined it with a genetic algorithm, that empowers AMP design targeted to a keystone pathogen. Predicted activity of the generated AMPs were classified using rough sets and the rules are improved using empirical growth inhibition data for specific pathogens. The validation tests were run with the peptides having the highest inhibition predictability score against the keystone pathogen, *A. actinomycetemcomitans*. A novel peptide VL-13 was confirmed to be active against the selected keystone pathogen without compromising the accessory-commensal species, *S. gordonii*.

## 2 Materials and methods

### 2.1 Materials

*A. actinomycetemcomitans* strain D7S-1, *S. gordonii* strain Challis, and *Streptococcus sanguinis* strain ATCC10556 were cultured using either modified Trypticase Soy Broth (mTSB) containing 3% trypticase soy broth and 0.6% yeast extract or on mTSB agar (mTSB with 1.5% agar (Becton Dickinson and Company). In some experiments, the bacteria were cultured in SHI medium supplemented with hemin (5 μg/ml) (Sigma-Aldrich, St. Louis, MO, USA), menadione (1 μg/ml) (Sigma-Aldrich), human serum (10%) (Sigma-Aldrich), and sucrose (0.25%). Bacteria were cultured at 37 °C in a humidified atmosphere supplemented with 5% CO_2_ (54). *P. gingivalis* strain ATCC33277 was cultured in the brain heart infusion (BHI) broth at 37 °C under anaerobic conditions.

### 2.2 Bacterial viability tests

Bacterial viability tests were done by first adjusting the optical density of bacterial cultures to 0.2 (equivalent to approximately 10^7^ CFU per ml) at 600 nm. The bacterial cultures were then diluted 1:20 to get to 5 × 10^5^ CFU/ml and incubated with 100 μM AMPs. Bacterial viability was then determined at 0 and 6 h by CFU counts and at an additional 24-h time point for *P. gingivalis*.

### 2.3 Machine learning model

#### 2.3.1 Initial datasets

The complete listing of the antimicrobial peptides used to generate the targeted rough set theory rules is given in Supplementary Table S1. In addition to literature-derived peptides, we included additional antimicrobial peptides previously studied for different applications shown in Table 1. The initial datasets for peptide generation were taken from the iAMP-2l database (55). This database was filtered to only include examples of anti-bacterial peptides, resulting in 1,274 unique peptides from the database. Of the 21 peptide sequences provided in Table 1 and Supplementary Table S1, 15 of them were already included in the database. Therefore, a total of 1,280 AMP sequences are included in the initial set.

#### 2.3.2 Peptide customization by rough set theory

We generated the rough set theory rules as described in our earlier publication on the CLN-MLEM2 method developed for classification of antimicrobial peptides with two enhancements (52). Previously, the rough set rules were designed to establish the boundaries simply between active and inactive antibacterial peptides, using non-correlated AAindex1 properties. The first enhancement we made is to combine the rough set rules from our previous paper with the targeting rough set theory rules and generate a multiple-dimensional view of the predicted activity. To generate these targeted activity rules, a set of AMPs with confirmed antibacterial activity against any of the three pathogens of interest, i.e., *P. gingivalis, S. gordonii, A. actinomycetemcomitans*, were used as positive data set (see Table 1, Supplementary Table S1 and Figures S1–S3). The second enhancement for targeting was introduced by focusing on the key physicochemical property features. We integrated eight of indices proposed by a recent study as reduced AAindex (rAAindex) obtained from a subset of original 544 original indices in the amino acid index database (56). Kibinge et al., applied a random forest (RF) algorithm for property reduction and maximizing metadata capturing. With the two-enhancement introduced; our method creates collections of discriminating attributes separating targeted AMP activity trends focused on hydropathy variations for peptide sequences.

The resulting rules are each characterized by a hydropathy property of importance determined by the updated CLN-MLEM2 method, and a simple summary characteristic that portrays the features to be used to predict an AMP`s targeted ability. Sequence features that are most relevant for the observed peptide activity are collected as simple arithmetic summaries. The summary characteristics used included the sum of the property across the amino acids of the sequence, the mean of the property across the amino acids of the sequence and the maximum value of the property across three consecutive amino acids within a sequence (50). These summary characteristics correspond to nonlinear boundaries between activity classes. Each time a rule set is generated, these boundaries and properties are chosen to separate the sequences into the desired classification groups. The heuristic goal of the method is to create definitions of activity classification with the minimum number of rules and conditions per rule possible. Rule sets contain descriptions of active and inactive peptides, but likely do not contain the set of all peptides in the union of the rule sets. Peptides which do not meet any of the rule sets are not identified directly by our classification method. Since specific peptide activity is not likely to be present for randomly selected sequences, we impute peptides as inactive if they do not belong to any rule set positive for activity.

#### 2.3.3 Sequence expansion by codon-based genetic algorithm

We used the codon-based genetic algorithm for sequence expansion reported in one of our earlier publications (53). To begin this process, we use a total of 1,280 AMP sequences in the initial set, which contained a large variety of sequences to recombine and mutate through artificial genetic operations. These sequences were subsequently ranked by which generated sequences met the rough set theory rules for targeted antimicrobial activity. The rule sets have two separate descriptions of antimicrobial activity in this study. The first description is the rough sets previously used as a measure for broad-spectrum activity estimation. The second description is the newly generated rules targeting the chosen periodontal keystone pathogens. This second description level of activity distinguishes between keystone-only activity and other antimicrobial activity with the goal of avoiding impacting commensal species. These two descriptions were weighed as components of the fitness function. There is a large disparity between the number of previously trained sequences, i.e., 2,347 sequences, and the empirically tested targeted sequences, i.e., 21 sequences (Table 1 and Supplementary Table S1). The rule counts were therefore independently calculated and normalized before being combined as separate terms in the fitness objective function. We defined fitness objective function for the codon-based genetic algorithm by the following equation in which *AB* is referred as antibacterial:

(1) F(x)=min(10*(MaxTargetRuleCount-SequenceTargetRuleCount)+0.01*(MaxABRuleCount-SequenceABRuleCount)+5*min([10,30]-SequenceAminoAcidLength))

The mutation rate for sequences to go to the next generation is 25%. Mutation changes a codon in the sequence, which may not result in an amino acid change or could result in a stop codon. The crossover rate was 50%. Crossing over was completed with codon representation, often resulting in frameshifts for new candidate sequences compared to the parent codon sequences.

The generations were monitored for convergence, both with the best fitness between generations and the consistency of the targeting rules to generate the top-scoring sequences. Generations are deemed mature for identifying top candidates when the majority of sequences meeting the targeting rules are within one half of the maximum number of targeting rules.

#### 2.3.4 Peptide synthesis

Peptides were synthesized using Wang resin following a standard Fmoc chemistry method using an Aapptec Focus XC peptide synthesizer. Dimethylformamide (DMF) and 20%–40% piperidine in DMF was used for Fmoc deprotection with repeating twice. The peptide-resins were then washed with DMF. Activation of 0.2 M amino acids/DMF (2 equivalents) was performed by addition of 0.2M 2-(1H-benzotriazol-1-yl)-1,1,3,3-tetramethyluronium hexafluorophosphate (HBTU)/DMF. The coupling step was completed twice, and the procedure was repeated until the complete peptide was assembled on the solid resin support. Following synthesis, the peptide-resin was removed from the reaction vessel using DMF. Following the removal of DMF from the peptide-resin by washing with ethanol, a cleavage cocktail (15 ml/1 gram of resin) was added to the dried resin for 2 h with gentle stirring to remove the peptide from the solid support and remove the side chain protecting groups. The standard cleavage cocktail is composed of trifluoroacetic acid (TFA)/triisopropylsilane (TIS)/water (95:2.5:2.5, % vol/vol/vol). To remove side chain protecting groups from peptides containing histidine or cysteine, 2.5% thioanisole and 2.5% 1,2 ethanedithiol were added to the cocktail and for peptides containing methionine, tyrosine, or arginine, 5% phenol was added. The cleavage products were filtered, and crude peptide product was isolated by precipitation in cold ether. The crude peptide was pelleted by centrifugation (2,000 rpm for 2 min), the supernatant was removed, the process is repeated for two to four times prior to lyophilization of the peptide products.

## 3 Results

**FIGURE 1:**
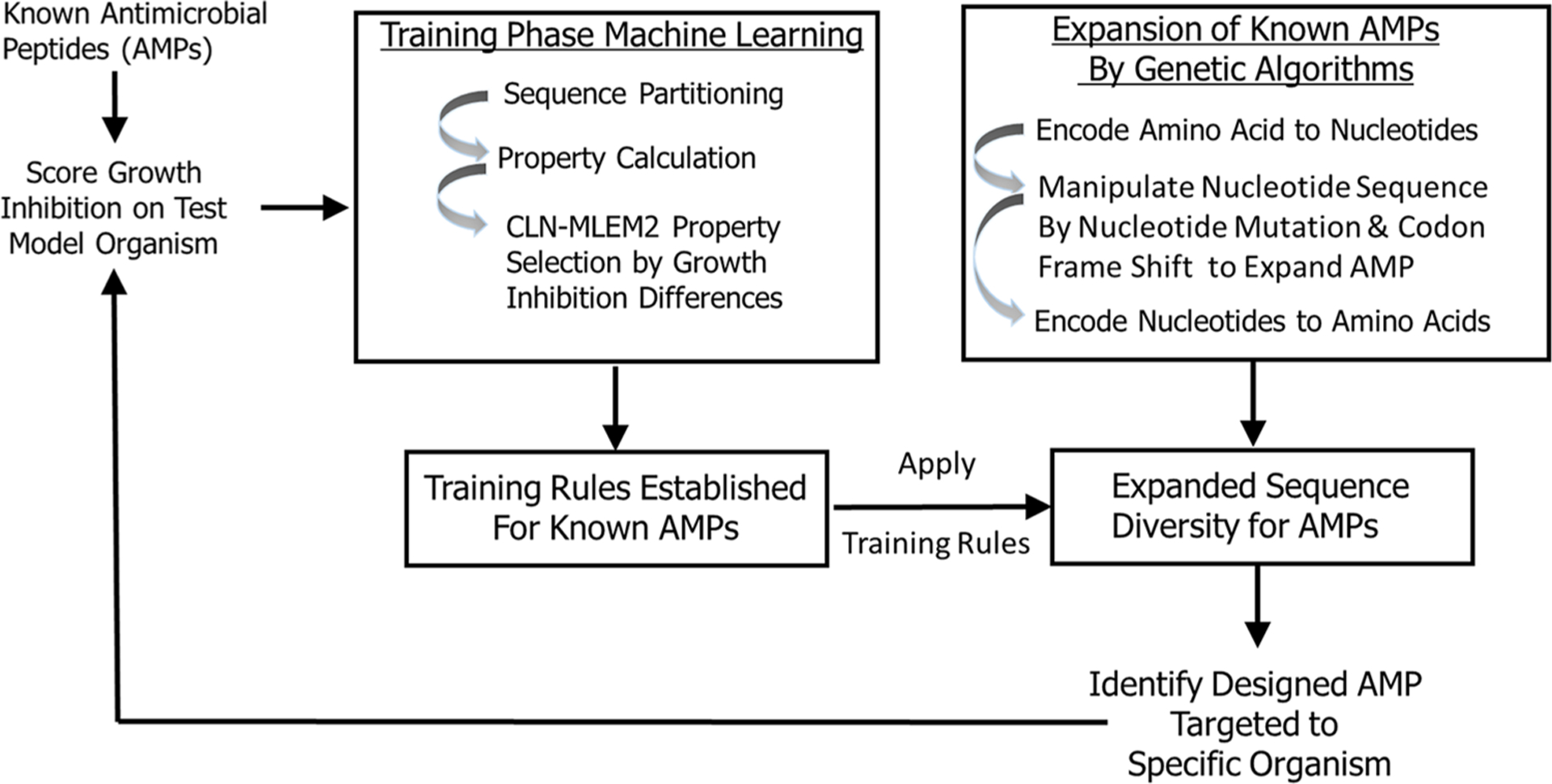
Peptide targeting design scheme for antimicrobial peptides. The training based on peptide sequences with identified growth inhibition. The expanded sequences are selected for consistency with identified training relationships.

In this study, we developed a transparent ML model that allowed us to incorporate iterative training sets and enriched our sequence space by a genetic algorithm to design antimicrobial peptides with inhibitory activity targeted to periodontal keystone and accessory pathogens. To identify antimicrobial peptides specific to oral pathogens, we first established rough set boundaries for training rules, building upon our initial iteration of known AMPs and their antimicrobial activity (Supplementary Figures S1–S3, Table 1, and Supplementary Table S1). Our rough set theory classifier was trained to identify possible antimicrobial peptides specific to oral keystone and supporting pathogens, with separate rule sets for each strain. We expanded sequence diversity using a genetic algorithm and selected novel candidates consistent with their predicted inhibitory activities using the identified training relationships. These candidates are generated by the second iteration. While the rough set rules generated apply to many other sequences than the sequences we trained on, the rules will not, in general, cover all sequences. Non-conforming sequences are imputed as non-targeted. Therefore, our second iteration is focused on finding the sequences which are like the sequences we identified as active against a targeted species in the first iteration considering the feature properties found to be discriminating between active and inactive against that single species. Training sequences do not need to have specific activity to generate sequences with targeted activity; multiple examples of non-specific peptides can still provide direction for what features are needed in generating rules. Figure 1 provides the selectively targeting antimicrobial peptide design scheme which includes training the next iteration of our model with known AMPs, establishing training rules, expansion of sequence diversity, candidate selection and verification of their predicted inhibition properties targeted to specific organisms. The targeting rules are heuristically made to be a minimal set which covers all our training sequences.

### 3.1 Establishing boundaries for growth inhibition rules

Our machine learning approach builds upon our previously developed CLN-MLEM2 method which utilizes rough set theory principles (52). The CLN-MLEM2 method separates sequences by their amino acid properties to establish functional classification. This method simultaneously filters which properties are key properties and provides boundaries for classification. The key properties identified by the CLN-MLEM2 method are provided in Supplementary Table S2. These properties were selected from the rAAindex (56), a reduced subset of the AAindex focused on hydropathy. We initiated the machine learning training by providing literature data to set up the initial inhibition descriptions for rough set participation to address selected pathogens (Supplementary Table S1). The rough sets are key property summary descriptions (e.g., property sum, property mean, property peak window) that identify peptides to have a certain activity level. The key property summary descriptions are used as features in our ML method (57). Both the full sequence length properties and short sequence segments are included as features. The short segments are summarized for a sequence by selecting the property peak window features. The full-sequence length features are included as the property sum and the property mean values. The CLN-MLEM2 method used 6 of the 8 properties in the rAAindex to build the rule sets. The selected properties and their short descriptions are included in Supplementary Table S2. The inhibition activity data captured from the literature was also supplemented by incorporating the inhibitory activity from additional antimicrobial peptide sequences into the model. These sequences have been shown to be active in different contexts, therefore we evaluated their activity against selected oral pathogens, *P. gingivalis*, *A. actinomycetemcomitans*, and *S. gordonii* (see Supplementary Figures S1–S3). The level of minimum inhibitory activities is categorized from low to high and provided in Table 1.

The ratio of correctly identified cases to the total number of applicable cases for a set is identified as *α* (0 ≤ *α* ≤ 1) (58–60). The CLN-MLEM2 method selects rules based on *α* (0 ≤ *α* ≤ 1). Using higher values of *α* generates fewer rules with higher probability (Pr) values of training accuracy if generated rules do not meet the accuracy specification. Using lower values of *α* generates more rules with lower Pr values of training accuracy when rules do not meet the accuracy specification. For all our training peptide sets, we found every rule with *α* = 1.0. Therefore, all generated rules meet the maximum accuracy specification of 1.0. Our current descriptions allow for enough discrimination to uniquely describe all peptide sequences when they differ in activity. We found hydropathy feature boundaries which explicitly classified our training examples. We next generated the CLN-MLEM2 rules for predicting the activity against keystone pathogen members *A. actinomycetemcomitans* and *P. gingivalis*, and accessory-commensal class member *S. gordonii*. These rules provide design criteria for either increasing or decreasing the probability of a peptide sequence having an antimicrobial activity against each strain. Our initial design strategy involved finding an antimicrobial peptide which heuristically has as many features as possible to be active through our genetic algorithm to satisfy the maximum count of non-linear boundary rules simultaneously through computational search.

Table 2 shows the selected sequence property rules that are associated with the inhibition of *A. actinomycetemcomitans* and *S. gordonii*. The sequence summary features in this table provide conditions for classifying peptides with potential inhibitory properties. We provide a detailed analysis of the *A. actinomycetemcomitans* inhibition rules in Supplementary Figures S4–S9. We found that the rules positive for *A. actinomycetemcomitans* inhibition are consistent with the first iteration identities and apply to either slightly polar mean polarity or to slightly non-polar mean polarity sequence descriptions, depending on the scale used. Interestingly, instead of the descriptions being mutually exclusive, VL-13 combined membership of both rules into a single peptide sequence.

### 3.2 Ranking antimicrobial peptides by rough set theory relevance

**FIGURE 2:**
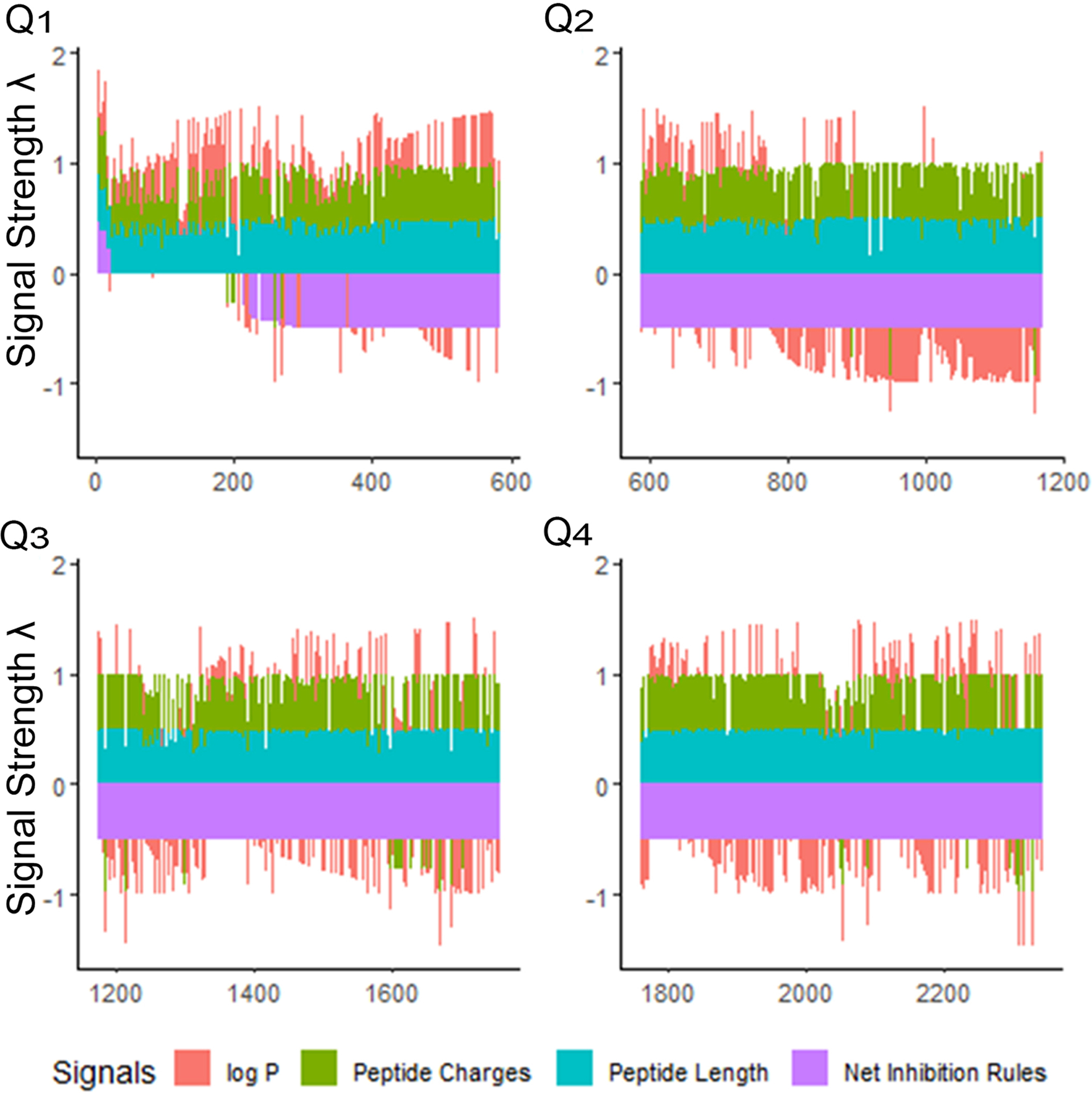
Peptide signals for iAMP-2l antibacterial sequences and training sequences (2,347 peptides). The signals are stacked columns of four different signals: log *P*, peptide charge, peptide length and net inhibition rules. The height of the stacked signals is limited to 0.5 using the inverse logit of the signal value, normalized as percentage of the overall observed range for the value. The database is divided into quartiles (Q1–Q4) of decreasing fitness. This view indicates immediately how many peptides have positive log *P* (hydrophobic), and which have negative log *P* (hydrophilic), in the order of the fitness criteria. The fitness criteria prioritize sequences having positive net inhibition rules for targeting. Priority is also given to shorter, more hydrophilic peptides for easier synthesis. Signal strength *λ* is calculated to maximize the sensitivity at the lower percentile ranges of property values. See Supplementary Materials 1.1.

Once “rough set” boundaries are established from our first iteration, antimicrobial peptides in the database can be compared in relation to the selected physical properties of their amino acids. We explored the selectivity of our training methods for the iAMP-2l database, which included 2,347 peptides (55). Using the CLN-MLEM2 rules, we ranked this database and generated a relatively small number of sequences of interest for further exploration (Figure 2). After combining the peptides in the iAMP-2l database and our initial testing set, only 20 peptide sequences (0.9%) met conditions for CLN-MLEM2 rules, the net of which were rules for inhibition instead of non-inhibition.

Overall, only 20 peptides in the iAMP-2l database (~2,200 peptides) had similar hydropathy features to the confirmed active AMPs and thus met the CLN-MLEM2 rules derived from our first iteration in antimicrobial activity prediction. We avoided selecting peptides with cysteine to enable rational cyclization studies using disulfide bonds in the future. Therefore, the third-ranked peptide KF-18 (KWKLFKKIPKFLHLAKKF) was selected directly from the database to synthesize and to evaluate its *in vitro* activity. To our knowledge, inhibitory activity for this peptide is not reported for oral bacteria.

### 3.3 Identifying candidate antimicrobial peptides with enhanced relevance to existing rough sets

We recently developed a codon-based genetic algorithm (53) to identify antimicrobial peptides relevant to inhibition-related rough sets, as well as no growth inhibition rough sets. Our codon based-genetic algorithm uses reading frameshifts probabilistically to generate new amino acid sequences that have low sequence similarity to previously generated sequences. The codon-based operations supplement the recombination and mutation operators. We targeted peptides identified as possessing antimicrobial properties for at least one of three target bacterial strains. We further ranked the peptides by length and solubility estimates.

**FIGURE 3:**
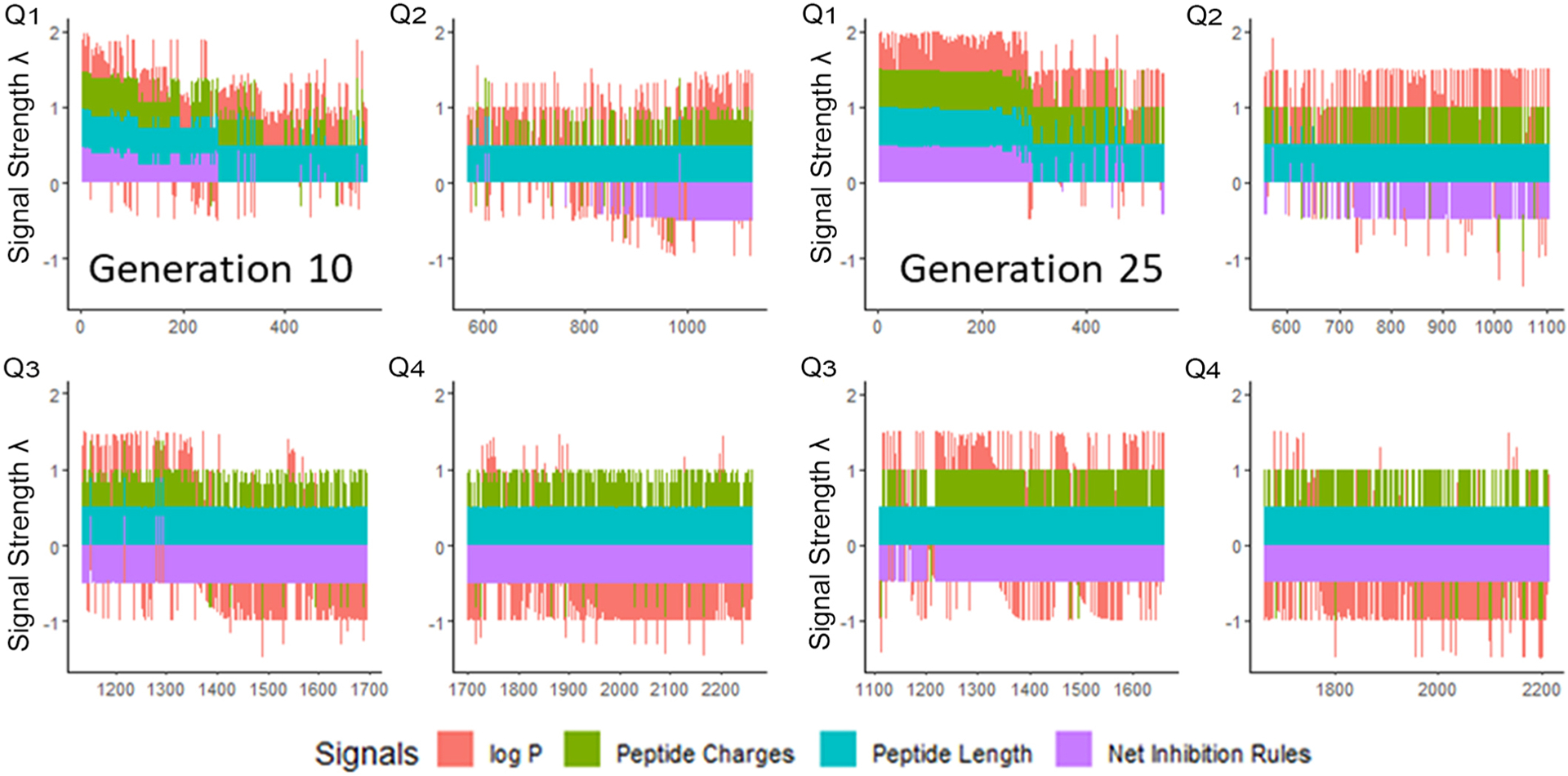
Peptide fitness signal quartiles (Q1–Q4) for 10th and 25th generation sequences. The initial generation seen in Figure 2, has only 20 sequences in the first quartile which meet the targeted inhibition rules. From this small set, the genetic algorithm generated peptides which have >200 sequences meeting the inhibition rules by Generation 10. However, the variation of the net inhibition rules for Generation 10 is high among these peptides. By Generation 25, maturation is achieved with >300 sequences with low variation of net inhibition rule conditions. Signal strength *λ* is calculated to maximize the sensitivity at the lower percentile ranges of property values. See Supplementary Materials 1.1.

**FIGURE 4:**
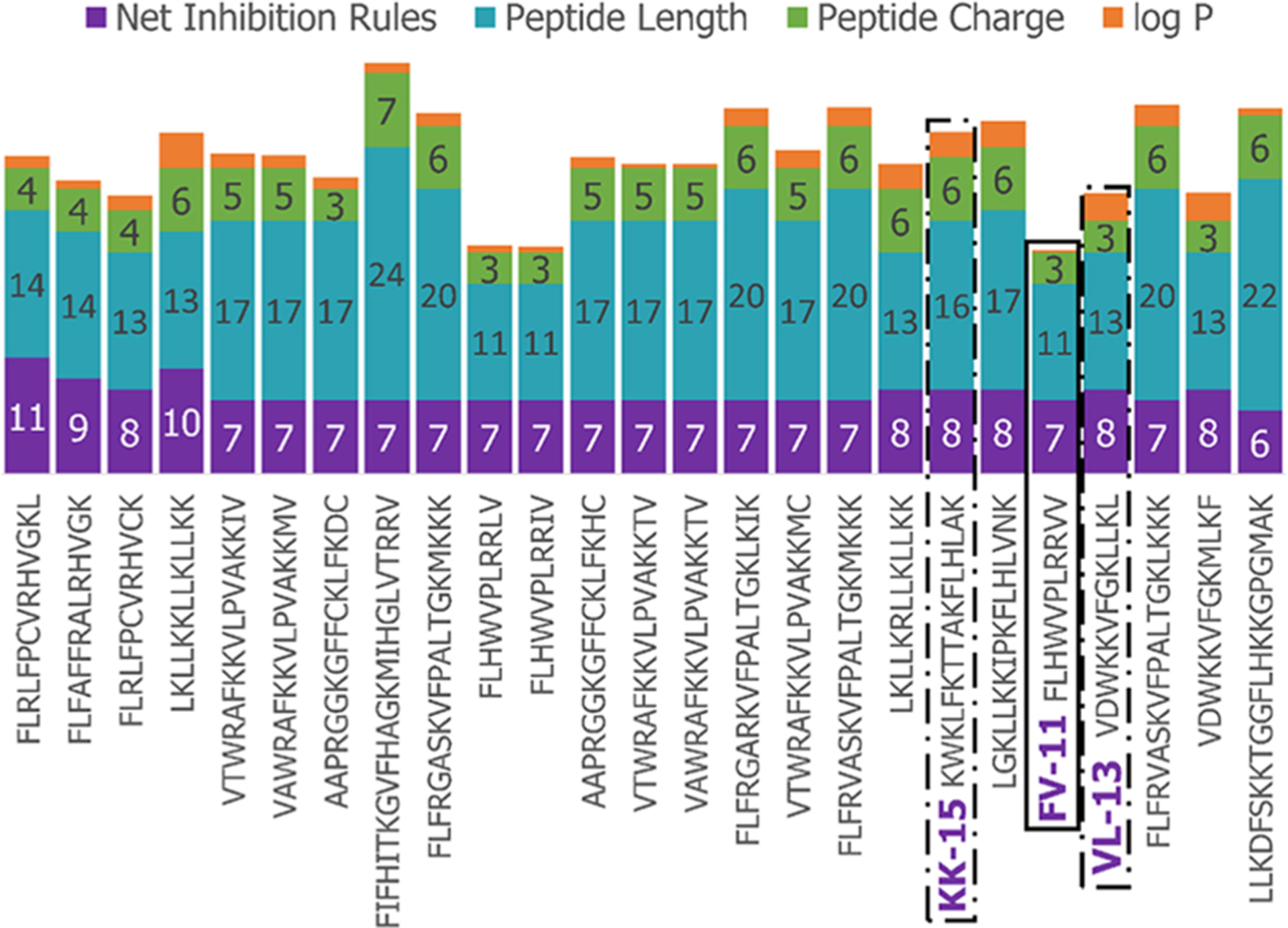
Peptide property values for Top-25 scoring sequences in the 25th generation of 2,261 sequences. The candidates selected for further experimental validation are highlighted in purple: KK-15, FV-11, and VL-13.

We next enriched the later-generation peptide sequences with sequences which met our rough set criteria. In Figure 3, this enrichment can be seen in the net-positive inhibition rule sequences where the initial generation has increased from 20 sequences (Figure 2) to 269 sequences by Generation 10. Generation 25 has a tighter distribution of net inhibition rules compared to Generation 10, which resulted in 329 peptide sequences. These sequences also contained the maximum number of net inhibition rules observed with the top scoring sequences are transferred between generations. The end of 25th generation run resulted in 2,261 sequences, from these we selected three peptides to evaluate their inhibition activity: KK-15 (KWKLFKTTAKFLHLAK), FV-11 (FLHWVPLRRVV) and VL-13 (VDWKKVFGKLLKL) (Figure 4). Many of the top scoring sequences contained cysteine and they were avoided to enable future cyclization of candidates through rational placement of disulfide bonds. We chose to have a high number of rules (>7), which were satisfied by KK-15 and VL-13. We also did not select FLFAFFRALRHVGK (FK-14), LKLLKRLLKLLKK (LK-13–1) and LKLLKKLLKLLKK (LK-13–2) peptides. The LK-13 and LK-13–2 peptides were observed as close analogues of AMP1, only missing the last lysine residue or also having an arginine-lysine substitution. Since AMP1 is already included in the study, we selected other candidates. Peptide FK-14 was not selected because it has a GRAVY score of +0.72, indicating a solubility risk. However, we selected the shorter peptide, FV-11, because it has less of a solubility risk with a high score of 7. As a summary, we focused our attention on potential solubility and possessing the most applicable rules. These candidates were among the top 25 sequences with 13 amino acids or less that fell within the 98th percentile of net inhibition rules. The maturation of generating new candidates with similar net inhibition rule counts was completed by the 25th generation. Noteworthy is the finding in Figure 3 that the 11th generation shows large variations of net inhibition rules among the candidates.

**FIGURE 5:**
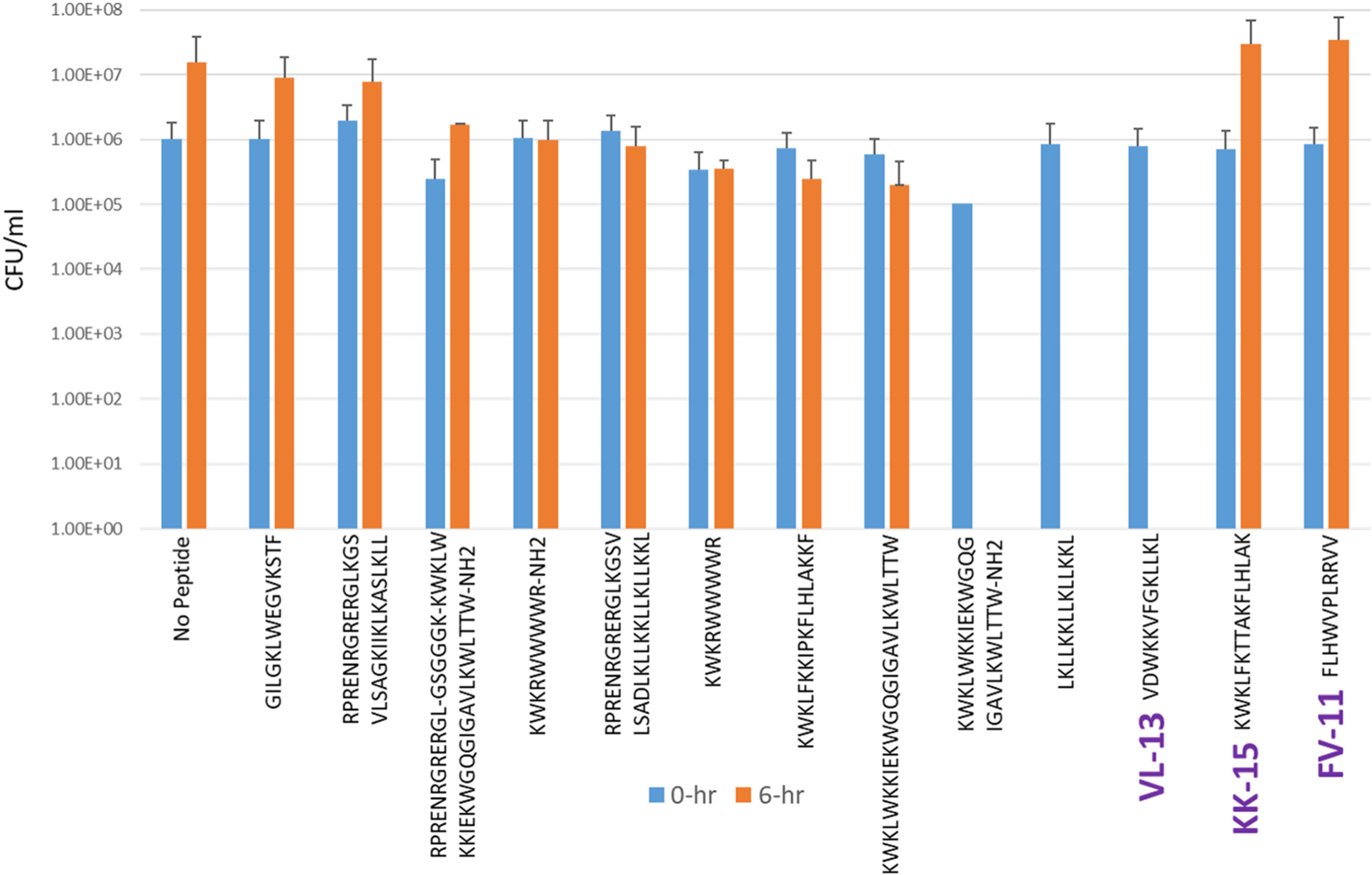
The *in vitro* analyses of ML generated top scoring peptides: VL-13, KK-15, FV-11 against keystone pathogen *A. actinomycetemcomitans*. Peptides without 6 h columns had no countable CFU/ml. The other included peptides are antimicrobial peptides which have known activity outside of the oral environment. Keystone targeting score is the difference in change in CFU/ml of the keystone pathogen and the minimum inhibition for either commensal strain. The keystone targeting scores are in Table 3.

**FIGURE 6:**
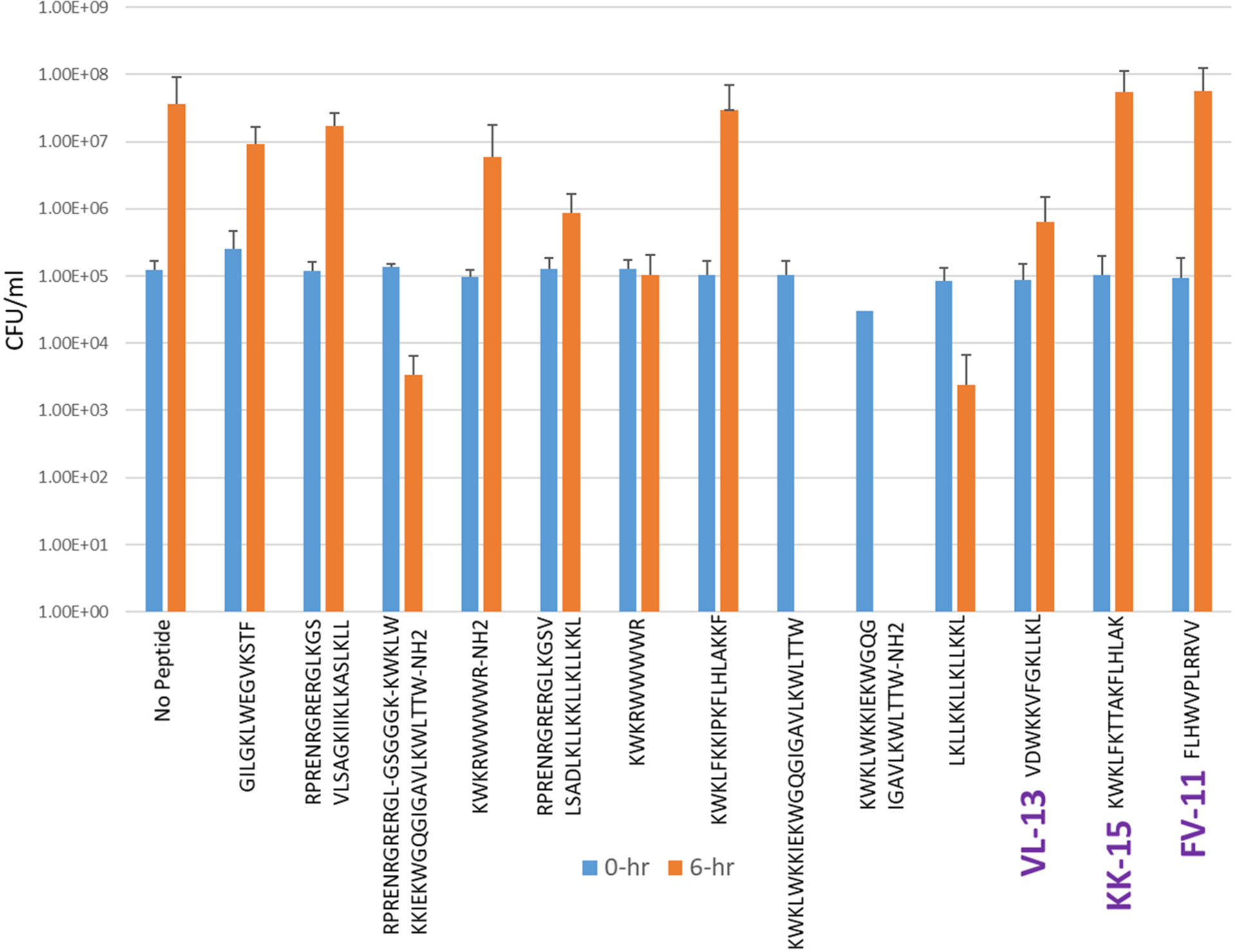
The *in vitro* analyses of ML generated top scoring peptides: VL-13, KK-15, FV-11 against accessory pathogen *S. gordonii*. Peptides without 6 h columns had no countable CFU/ml. The other peptides are antimicrobial peptides which have known activity outside of the oral environment. Keystone targeting score is the difference in change in CFU/ml of the keystone pathogen and the minimum inhibition for either commensal strain. The keystone targeting scores are in Table 3.

**FIGURE 7:**
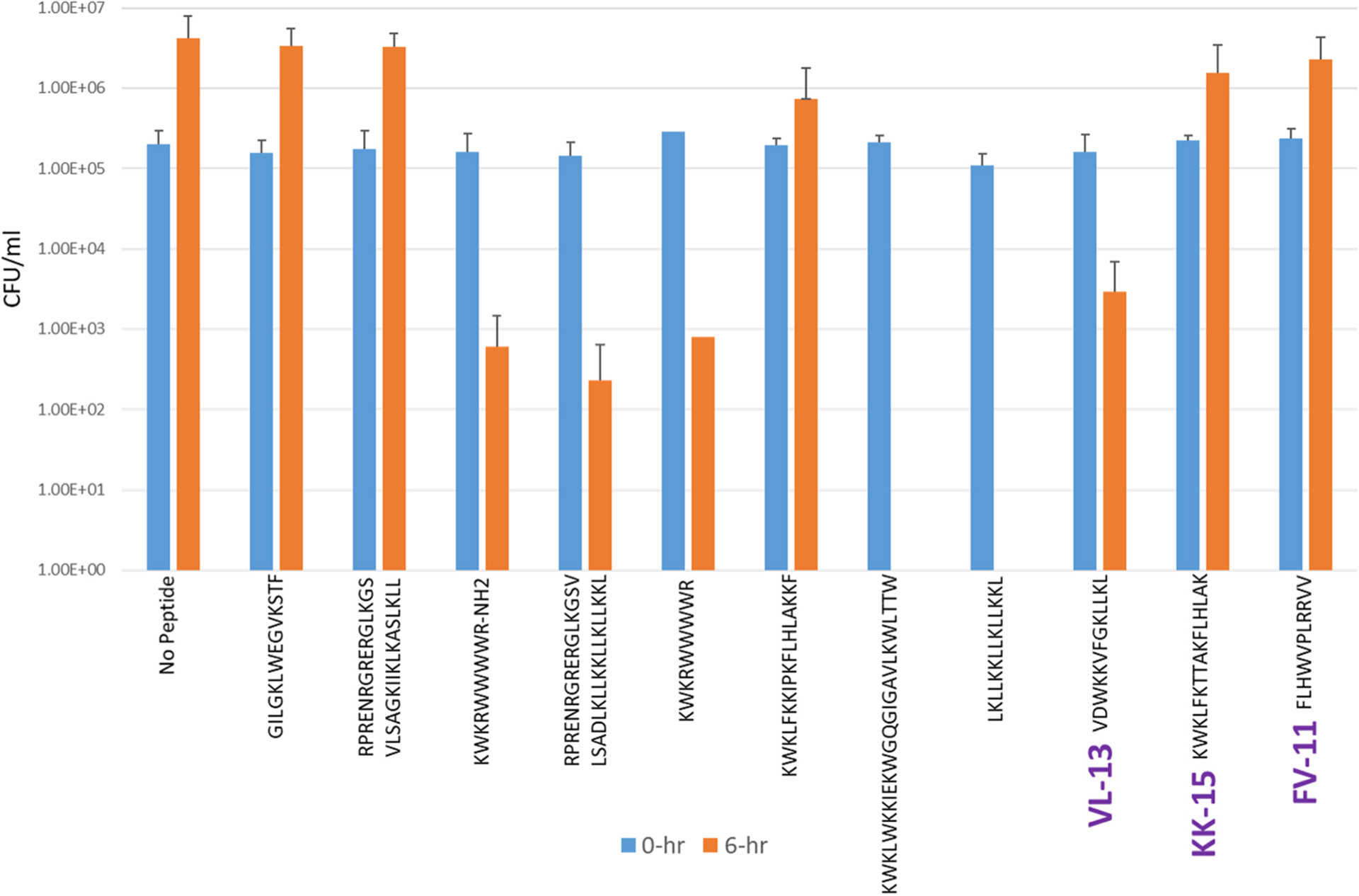
The *in vitro* analyses of ML generated top scoring peptides: VL-13, KK-15, FV-11 against commensal strain, *S. sanguinis*. Peptides without 6 h columns had no countable CFU/ml. The other peptides are antimicrobial peptides which have known activity outside of the oral environment. Keystone targeting score is the difference in change in CFU/ml of the keystone pathogen and the minimum inhibition for either commensal strain. The keystone targeting scores are in Table 3.

Experimental evaluation of ML generated sequences, KK-15, FV-11, and VL-13 were performed against keystone pathogen *A. actinomycetemcomitans*, accessory/commensal *S. gordonii* and commensal *S. sanguinis* (Figures 5–7, respectively). We compared these second iteration peptides with two first iteration peptides, AMP1 and AMPa. Table 3 shows the scores for targeting keystone pathogens. VL-13 showed strong inhibition activity selectively against *A. actinomycetemcomitans* compared to the other peptides FV-11, KK-15, and KF-18. The score for targeting keystone pathogen is the difference between the number of CFU logs reduced after 6 h. VL-13 has the largest targeting score, having +7 more log reduction for the keystone pathogen *A. actinomycetemcomitans* than for the commensal *S. gordonii*. VL-13 also had a high score for targeting keystone pathogen of +4.5 when compared to the other commensal strain *S. sanguinis*. The other predicted peptides had positive targeting scores, but less than AMP1. Peptides used in the training sets AMPa resulted in the lowest targeting score of only a 0.5 log reduction difference between the two groups; AMP1 also resulted in activity against *S. sanguinis*. VL-13 was validated for predicted targeted activity against *A. actinomycetemcomitans*.

**FIGURE 8:**
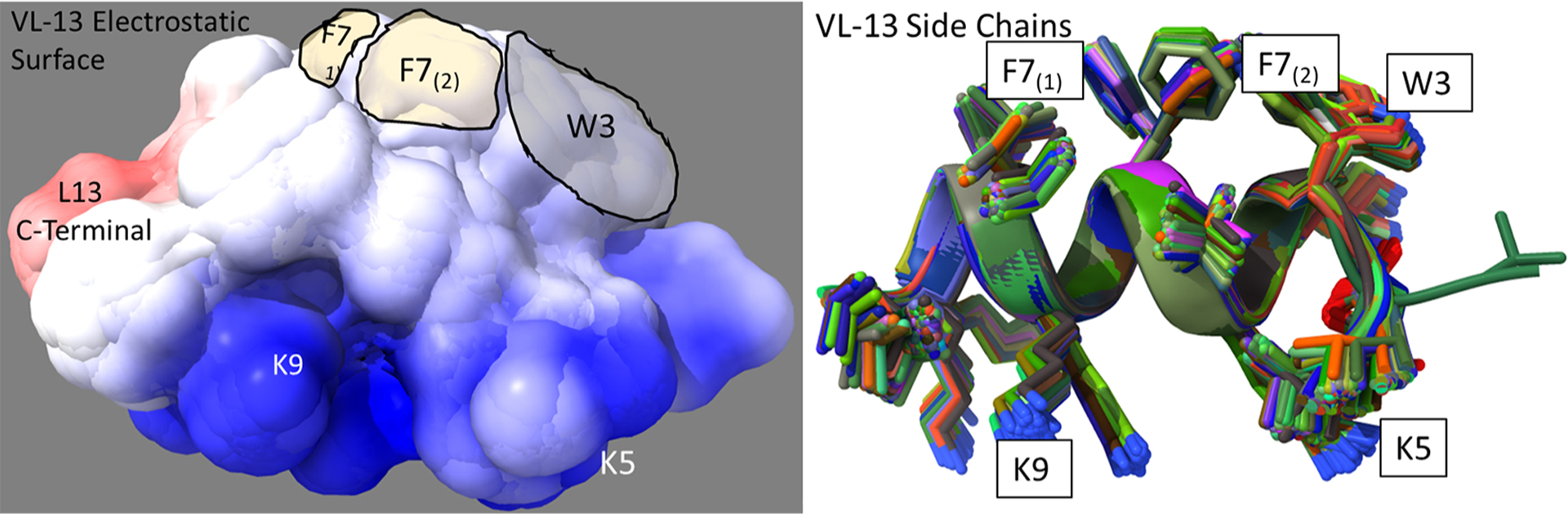
Ensemble structure description of VL-13 (VDWKKVFGKLLKL). The hydrophobic feature of VFG (see Figure 10) has very high solvent accessibility. This indicates high accessibility for potential binding partners. The locations of the side chains are stable through the ensemble. Only the Phenylalanine 7 seems to have multiple distinct clusters of orientations, which may indicate a local rotation between two smaller vibrations of the aromatic ring. The conserved backbone structure simplifies structure-function hypotheses for peptides. This lower folding entropy simplifies structure-function studies compared to using AMPa or AMP1. Superposition of 192 PEP-FOLD3 (66) structures aligned with MatchAlign (64) in UCSF Chimera (65).

**FIGURE 9:**
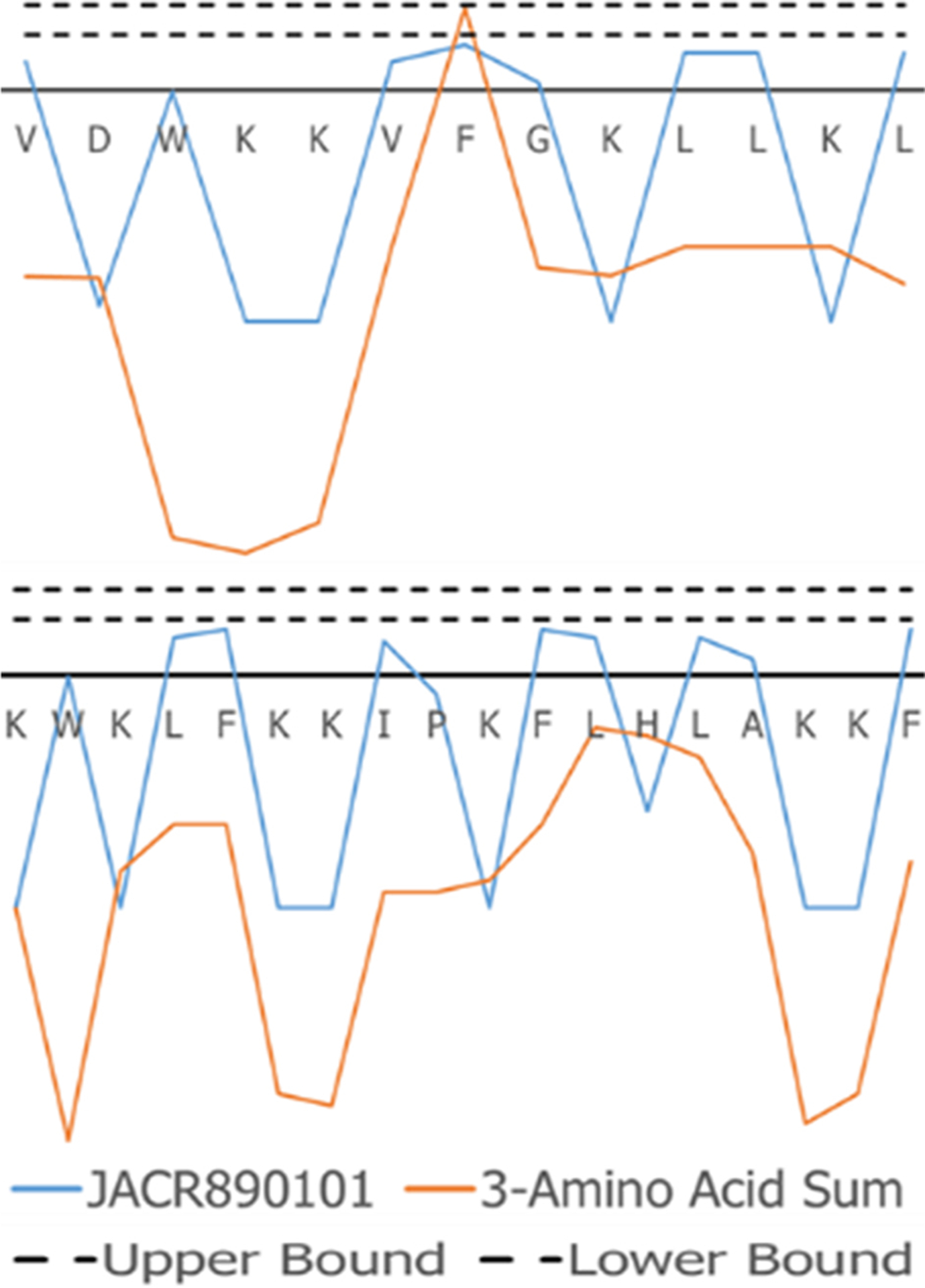
JACR890101 hydrophobicity property trend as both a single amino acid property (blue) and as the sum of three consecutive amino acids (orange). In (**A**), VL-13 meets Rule 1 Condition 2 in Table 2 at residue F7 where the 3-aa window falls within the rule bounds (dashed lines). In (**B**), KF-18 does not have a hydropathy feature of the 3-aa sum that meet this condition. The dashed lines indicate the Rule 1 Condition 2 upper and lower boundaries in Table 2. Figure S4 compares Rule 2 and Rule 3 conditions in Table 2 with Rule 1.

The superimposed structures generated for VL-13 are given in Figure 8. From the structures, a peptide structural feature with a high amount of hydrophobicity is indicated as a low-energy rotation barrier compared to the more hydrophilic structural features in the antimicrobial peptide. This hydrophobicity feature is also discussed in the rough set theory sequence feature example with the JACR890101 3-amino acid window in Figure 9 and in Supplementary Figure S4.

As a parallel method to assess the progress of the CLN-MLEM2 model, we predicted the performance of additional peptides reported as potential therapeutics for oral bacteria and oral biofilms as the test set (61). In Supplementary Tables S3 and S4, we evaluate the performance of the rules specific to two keystone pathogens, *A. actinomycetemcomitans* and *P. gingivalis*, respectively. In Supplementary Table S5, we evaluate the performance of the rules specific to the commensal/accessory pathogen *S. gordonii*. The false discovery rate was found to be low for *A. actinomycetemcomitans* and the commensal pathogen, *S. gordonii* (Supplementary Tables S3 and S5) using prediction performance. Overall, the model performance shows good prediction for positive activity of the sequences for selected keystone pathogen and the commensal/accessory pathogen.

## 4 Discussion

### 4.1 Shifting focus toward pathogen *A. actinomycetemcomitans*

Treatment of periodontal and peri-implant diseases necessitate a targeted, polymicrobial approach that sufficiently inhibits progression of the disease state without detrimentally impacting commensal and otherwise opportunistic species. To address this, we attempted to develop a ML enabled targeting AMP prediction approach. To train the CLN-MLEM2 model, an initial round of AMPs was tested against keystone pathogens *P. gingivalis* and *A. actinomycetemcomitans*, and opportunistic pathogen *S. gordonii*. Both *P. gingivalis* and *A. actinomycetemcomitans* display pathogenic characteristics that contribute significantly to biofilm prevalence and virulence (11). Although these species are referred as key stone pathogens, their involvement appears in different stages of the disease progression. The ML based tunability in this paper presents an opportunity to control the disease progression by targeting different keystones and other microbiome components.

*A. actinomycetemcomitans* is unique for its association with localized, aggressive cases of peri-implantitis and periodontitis —specifically those in younger individuals under the age of 35 (13, 62). This is extremely problematic for not only the livelihood of impacted individuals, but also for the whole of human health. Disease occurrence in younger individuals entails longer timelines of recurrent infection and treatment cycles. This makes these individuals, and thus bacterial species present in them, prime candidates for emergent bacterial resistance and innate microbiome depletion. It also makes them at heightened risk for implant installation failure and loss of oral functionality as the tissue and bone surrounding their dental implants degrade. In our ML predicted novel peptide generation, we therefore focused on assessment of the antimicrobial activity against *A. actinomycetemcomitans, S. gordonii,* and the commensal *S. sanguinis*.

### 4.2 Rule application and predicted vs. experimental antimicrobial activity in VL-13

The rules in Table 2 show amphipathic descriptions of targeting growth inhibition of *A. actinomycetemcomitans*, both for descriptions of which peptides inhibits and which peptides do not inhibit growth. In Supplementary Figures S4–S9, we describe the feature characteristics for sequences which also have rule membership for rules which apply to VL-13 in Table 2. Our second-iteration decision system made incorrect predictions for KK-15 and FV-11. The next iteration of the decision system will avoid these incorrect predictions and generalize with these new cases to determine new decision boundaries. The next decision system draws more accurate boundaries for negative results but does not move boundaries when all cases within a sub-domain are accurate. This attribute of our transparent decision system shows the system development value of having test cases. This way the decision system challenges the rough set membership boundaries of previous iteration rather than having test cases which are very likely to be accurate or peptides which have no rough set theory membership. Testing truly random peptides which do not belong to either rules for or against activity may not build on the knowledge gained from previously tested peptides. However, any peptide test results would start a new knowledge base to build on in future studies.

We used a nested-rough set rules approach by evaluating the rules generated when classifying peptides for having any antibacterial activity with rough set rules for peptides having targeted activity. This nested methodology is an example of transfer learning in our machine learning method. Further targeting of the activity of the peptides incorporating different design goals can also adapt this nesting approach in future studies.

In the first iteration of our codon-based genetic algorithm, generated a novel antimicrobial peptide against *S. epidermidis* (53). In our second iteration of this system in this work, we applied a nested version of our decision system to the *in vitro* oral environment for the mitigation of the progression of peri-implantitis. In this study, an antimicrobial peptide, VL-13, was demonstrated targeted growth inhibition against an oral keystone pathogen *A. actinomycetemcomitans* without inhibiting accessory/ commensal species, *S. gordonii* (Figure 5).

**FIGURE 10:**
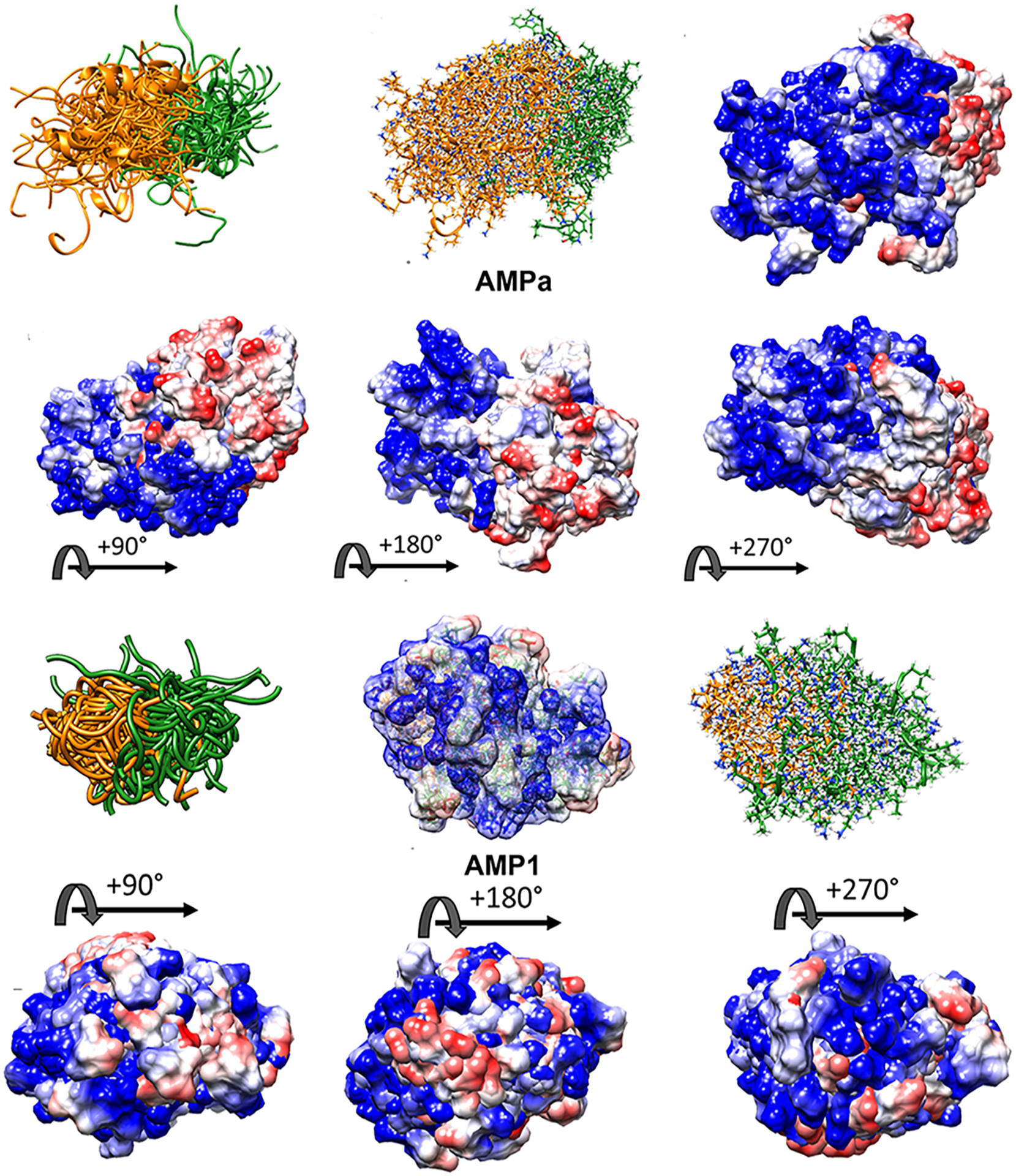
High folding entropy of AMPa (**A**) and AMP1 (**B**) compared to VL-13. Orange ribbon is the first half of each sequence and green is the second half of the corresponding sequence. The divergent ribbons indicate a folding ensemble with many different orientations of backbone structure having similar energy values. In Figure 9, the conserved backbone shape among superposition structures indicates a relatively large folding energy barrier to other backbone shapes. While the high structural entropy complicates the structure-function analysis of these peptides, surface interactions are still facilitated through electrostatic means. The electrostatic surface indicates highly charged surface which is mostly electropositive (blue) for the first half of AMPa with most electronegative (red) surface for the second half of AMPa. The distribution of electronegativity for AMP1 is more heterogeneous compared to AMPa. Superposition of 100 PyRosetta structures (63) aligned with MatchAlign (64) in UCSF Chimera (65).

VL-13 is both a positive result for this decision system iteration for activity against the keystone pathogen and a negative result for activity against the accessory/commensal pathogen *S. gordonii*. We designed peptides to meet multiple targeting classes, inferring that if the targeting criteria is relatively difficult to describe, finding one working targeting description would likely come before two working targeting descriptions. Our result does not limit building on the targeting criteria which could be introduced as new design characteristics. In future studies, we can use both experimental results to draw boundaries which include VL-13 for *A. actinomycetemcomitans* inhibition and exclude VL-13 for *S. gordonii* inhibition. Our method learns boundaries from inhibition and non-inhibition results for each of the tested peptides. Superimposed structures of AMPa and AMP1 were evaluated to gain further insight, as both sequences were used in the training set based on their demonstrated activity (Figure 10). We show that the folding dynamics of these structures have relatively high folding entropy compared to VL-13, shown in Figure 8.

To discuss what the rough set theory rules imply about inhibition activity for the keystone pathogens, we begin with one AAindex1 property. The 3-amino acid window JACR890101 is the amino-acid wise component of hydrophobic interactions of amino acids at the bilayer (Figure 9, Supplementary Figure S4). While further study can investigate if this feature is necessary for the peptide’s activity, we also note that this feature may be related to some motion allowing the peptide to efficiently attack/bypass the membrane of *A. actinomycetemcomitans*, which is a gram-negative pathogen. In Rules 1 and 3 in Table 2, this feature was selected as a tripeptide window or a mean (see Figure 10). The rules both select tripeptide windows for this feature, which Rule 1 having a left-shifted range (from 0.89–1.32 to 0.615–0.935). This window is very hydrophobic under the conditions of the bilayer (see Supplementary Figure S4–S9). The overall mean of the peptide for this property in Rule 1 was between −1.2 and −1.8, indicating that these tripeptide windows of greater than 0 are not likely to be common among peptides which are active against *A. actinomycetemcomitans*. The combination of these rules describes a preliminary description of amphipathy that is useful to inhibit the pathogen’s growth. Having varying hydrophobicity has long been studied for antimicrobial peptides (57, 67, 68). Further non-linear boundary ranges are shown in Supplementary Figures S1–S6. The CLN-MLEM2 rough set theory method has added a process to identifying which hydrophobicity features relate to inhibiting the growth for a specific pathogen. The target that the antimicrobial peptide is affecting with the hydrophobicity feature is unknown.

### 4.3 Testing performance, limitations and future perspectives

This paper used data from a review article on peptides as therapeutics targeting oral bacteria by Sztukowska et al. (61) as a test set. We recognize there exist previous reviews of antimicrobial peptides in the oral environment with peptide sequences that were not included in our training set (69–72). The reasons to not include all experimentally tested peptides in the model training phase is to continue to develop our model through testing performance beyond training performance. Having peptides in the literature that are not included in our training set allows for testing the performance evaluation of our model. Indeed, ML model should be validated with experimental results.

The literature peptide activity (61) was used as a test set for our rule sets describing targeted activity for the two keystone pathogens and the commensal/accessory pathogen. The first keystone pathogen rules for *A. actinomycetemcomitans* had higher accuracy than the commensal pathogen rules for *S. gordonii*, indicating a closer relationship between trained sequences and tested sequences by the rule set descriptions related to Supplementary Table S3 than to the rule set descriptions in Supplementary Table S5. The rule set for the second keystone pathogen had a high false discovery rate, indicating the rule set related to Supplementary Table S4 for *P. gingivalis* has a higher chance of leading to unexpected negative inhibitory results. These results also confirm the critical iterations with the enhanced rule sets for different pathogens including keystones such as *P. gingivalis*. Our future studies focus on developing rule sets leading to low false discovery rates (<10%), then we will incorporate this bacterial strain in our ML-candidate evaluation process. Our ML-models are re-trained between iterations to avoid carrying over recognized performance errors.

Hydrophobicity trends among peptides are discovered during training and applied to the selection of new peptides. Our method is identifying database peptides which have similar hydropathy features to the peptides that are confirmed for their antimicrobial activity using *in vitro* evaluation against our targeted bacterial strains. These activities build better information for peptides with similar hydropathy features, rather than using brute-force testing on all related peptides to see if their inhibition activity changes in a useful way between our targeted bacterial strains. Incorporation of new information from *in vitro* results strengthens our approach for either positive or negative results. We recognize the fact that the peptides studied here need to be further evaluated for their toxicity. Our future work will include experimental evaluation of the potential peptide toxicity as well as other clinically relevant properties of these peptides. We also plan to incorporate multiple factors into the ML design of targeted peptides. Broader types of data, such as toxicity and stability, can be tightly integrated together with inhibitory activity because each new factor will have its own rule sets to simultaneously constrain the sequences the genetic algorithm will target. These distinct descriptions can be integrated with the inhibitory activity descriptions by building separate rule sets and using our genetic algorithm to find examples which combine multiple rule sets for distinct descriptions. The transparent approach of rough set theory allows us to re-classify sequences based on new results in a short time without using GPU-parallelized computation. Therefore, retraining of the entire dataset is feasible when integrating data between sources and extending data sources.

During the training, hydrophobicity trends among peptides were discovered and applied to the selection of new peptides. Our validation tests were run on three top candidates, one of which had the highest inhibition predictability score against the keystone pathogen, *A. actinomycetemcomitans*. Our *in vitro* test results demonstrated that peptide VL-13 is an antimicrobial peptide with the largest change of inhibition compared between the keystone pathogen and the accessory-commensal species, *S*. *gordonii*. Further, VL-13 has an added advantage in possessing the largest change of inhibition between the keystone pathogen and the commensal species, *S. sanguinis*.

## 5 Conclusion

We developed a transparent machine learning model with an iterative *in vitro* experimental validation approach to design antimicrobial peptides that selectively target keystone pathogens believed to play critical roles during biofilm dysbiogenesis leading to progression of peri-implant disease. The transparency of our machine learning method allows us to compare the discovered relationships with trends in literature. The non-linear nature of the boundaries also provides for rapid learning between iterations of new sequences to explore. Through our transparent machine learning methods, we demonstrate the finding that antimicrobial peptide VL-13 inhibits the keystone pathogen *A. actinomycetemcomitans*, while the ML model also provided better learning descriptions for finding antimicrobial peptides inhibitory against the accessory pathogen *S. gordonii*. VL-13 was the most targeted antimicrobial peptide sequence we tested, with high levels of inhibition against *A. actinomycetemcomitans* and minimal impact on *S. sanguinis* and *S. gordonii*. The descriptions used to select the VL-13 sequence from our candidate sequence generation method were learned from tested peptides in this study combined with known sequences in the literature. From antimicrobial peptide sequences available in iAMP-2l antimicrobial peptide database, we chose KF-18 to test for activity. KF-18 has strong homology with AMPa, which we demonstrated is inhibitory toward *A. actinomycetemcomitans*. Since this peptide and a second generated candidate with homology to AMPa (KK-15) did not test as active, we have further insight into the features of AMPa which relate to inhibition activity for *A. actinomycetemcomitans* and *S. gordonii*. Future work aims to generate non-inhibitory sequences for commensal and accessory species, such as *S. gordonii*, while retaining inhibitory activity against keystone pathogens. Developing an engineering approach to iteratively discover targeted antimicrobials is a robust method for potential therapeutic treatment for peri-implant biofilm infections and thus to reduce the resulting host response of hyper-inflammation during disease progression. With increasing use of implants to replace missing teeth and support oral function, the number of patients suffering from peri-implantitis will continue to increase. It is critical to find targeted approaches to address this complex infectious disease. Antimicrobial peptides with selective bioactivity against keystone pathogens, while preserving commensals could be the next generation therapy that will respond to this urgent healthcare need.

## Supplementary Material

Supplementary file

## Figures and Tables

**TABLE 1:** Rough set theory identities for *in vitro* inhibition against *A. actinomycetemcomitans* (Aa), *S. gordonii* (Sg), and *P. gingivalis* (Pg).

Name	Sequence	*Aa* inhibition	*Sg* inhibition	*Pg* inhibition
AMPa	KWKLWKKIEKWGQGIGAVLKWLTTW	High	High	Low
AMP1	LKLLKKLLKLLKKL	High	High	High
AMP2-NH2	KWKRWWWWR-NH2	None	None	None
TiBP-AH-AMP1	RPRENRGRERGLKGSVLSALKLLKKLLKLLKKL	None	None	None
TiBP-AH-GL13K	RPRENRGRERGLKGSVLSAGKIIKLKASLKLL	None	None	None
AMP7	ESYKKML	None	None	None
AMP10	GILGKLWEGVKSTF	None	None	None
TiBP-S5-AMPA	RPRENRGRERGLGSGGGKWKLWKKIEKWGQGIGAVLKWLTTW	None	None	None

**TABLE 2:** A set of CLN-MLEM2 rules for *A. actinomycetemcomitans* (Aa) and *S. gordonii* (Sg).

Activity identity	Rule	Training sequence support	Condition	Calculation	AAindex1 property	Lower-bound	Upper-bound
Growth inhibition of *A.a*.	1	3	1	Mean	ZIMJ680103	17.09	22.99
			2	Tripeptide Window	JACR890101	0.615	0.935
			3	Sum	COWR900101	−27.82	0.27
			4	Mean	JACR890101	−1.743	−1.218
			5	Mean	COWR900101	−0.359	−0.001
Growth inhibition of *A.a*.	2	2	1	Mean	WARP780101	6.401	7.141
			2	Mean	MEEJ810102	3.227	7.278
			3	Tripeptide Window	FAUJ880110	2.0	5.5
			4	Mean	FAUJ880110	0.40	0.69
No growth inhibition of *A.a*.	3	11	1	Tripeptide Window	FAUJ880110	6.5	12.0
			2	Tripeptide Window	COWR900101	3.78	5.41
			3	Tripeptide Window	WARP780101	26.825	30.12
			4	Tripeptide Window	JACR890101	0.89	1.32
Growth inhibition of *S.g*.	1	43	1	Tripeptide Window	FAUJ880110	3.5	12.0
			2	Mean	FAUJ880110	0.69	2.0
No growth inhibition of *S.g*.	2	35	1	Tripeptide Window	LIFS790102	3.505	7.890
			2	Tripeptide Window	WARP780101	24.64	30.12
			3	Tripeptide Window	COWR900101	2.9	5.41
			4	Tripeptide Window	JACR890101	0.89	1.32

**TABLE 3:** Net inhibition rule counts by bacterial species.

Name	Sequence	Aa NC	Sg NC	Pg NC	Aa LR	Sg LR	Ss LR	Keystone targeting
AMPa	KWKLWKKIEKWGQGIGAVLKWLTTW	2	4	−15	5	4.5	ND	+0.5
AMP1	LKLLKKLLKLLKKL	2	6	2	6	2	5	+4.0
KK-15	KWKLFKTTAKFLHLAK	2	4	2	−1.5	−3	−1	+1.5
FV-11	FLHWVPLRRVV	0	4	3	−1.5	−3	−1	+1.5
VL-13	VDWKKVFGKLLKL	2	6	0	6	−1	1.5	+7.0

Positive scores in the “NC” columns indicate predicted antibacterial activity for the strain. Zero or negative scores in these columns indicate no activity predicted. The “LR” columns indicate the decimal log change after 6 h of incubation with the peptide indicated in Figures 5–7. Keystone targeting is the difference between the *A. actinomycetemcomitans* (Aa) log reduction and the minimum of the *S. gordonii* (Sg) log reduction or the *S. sanguinis* (Ss) log reduction. Higher log reduction scores indicate more growth inhibition. Negative log reduction indicates growth during incubation instead of inhibition. Higher targeting scores indicate better targeting performance. The first two rows are first iteration sequences with known activity used to compare the targeting performance of our second iteration peptides generated by the codon-based genetic algorithm. The *Aa* inhibition rules describe transferred activity for VL-13 but not for KK-15, while the *Sg* inhibition rules did not describe transferred activity in any of our second iteration peptides. NC, net count of inhibition rules, LR, log reduction and ND, not determined.

## Data Availability

The original contributions presented in the study are included in the article/Supplementary Material, further inquiries can be directed to the corresponding author.
