# Supplementary material for: Machine learning enabled design features of antimicrobial peptides selectively targeting peri-implant disease progression
Source: Front Dent Med. Author manuscript; Available in PMC 2024 Jun 6. (PMC11155447; doi:10.3389/fdmed.2024.1372534)
Supplement: Supplementary file [file NIHMS1996707-supplement-Supplementary_file.docx]

***Supplementary Material***

1. **Supplementary Data**

This document contains materials that supplement *Machine learning enabled design features of antimicrobial peptides selectively targeting peri-implant disease progression.* Within the following pages, additional figures and tables are provided to create a more comprehensive picture of the development and design of novel, targeted antimicrobial peptides (AMPs) via our unique machine learning models (CLN-MLEM2, CB-GA).

- 1. **Supplemental Figure and Supplemental Table Outline**

Multiple peptides against *P. gingivalis*, *A. actinomycetemcomitans*, and *S. gordonii*. This data was used in the training and tailoring of the CLN-MLEM2 method, as discussed in the main paper. Figures S4-S9 contain a series of graphical depictions of six hydropathy property values of AMP VL-13 within the rule set boundaries for these values. The figures delineate how VL-13’s summary characteristics in these properties correspond to its predicted growth inhibition for *A. actinomycetemcomitans*. An additional summary of the rough set theory follows these figures.

Supplementary Table S1 presents a compilation of literature-based AMPs known for their activity against several periodontal pathogens. This table was also used in training the CLN-MLEM2 model of the current study. Supplementary Table S2 provides short descriptions of the hydropathy properties identified by the CLN-MLEM2 method as defining physiochemical traits for targeted antimicrobial ability.

We also predicted the performance of the CLN-MLEM2 model on additional peptides reported as potential therapeutics for oral bacteria and oral biofilms as the test set (Supplementary Tables S3-S5). Our model did not train on this test set. The rules sets showed good predictability for the selected keystone pathogen and the commensal/accessory pathogen. The false discovery rate was found to be low for the keystone pathogen *A. actinomycetemcomitans* and the commensal pathogen *S. gordonii* (Supplementary Table S3 and Supplementary Table S5).

- 1. **Peptide Sequence Signals**

In Figure 3 and Figure 4, the attributes of many peptide sequences, counted on the horizontal axis, are displayed together. One stacked bar of peptide signals is one peptide sequence summary. Each signal is a normalization of the value of the property over the range of the peptides in the database. The equation for the signal is:

$\lambda= 0.5*inverse logit(50*\left( \frac{sequence value}{max. list value-min. list value} \right))$.

The factor of 50 increases the sensitivity of the signal values for small values in the range. A factor of 1 has maximum sensitivity at the 50^th^-percentile. Adjusting the factor to 50 focuses the maximum sensitivity at the first percentile of the range. This characteristic makes this chart tailored to show the difference between differences in properties at low percentiles and less sensitive to show differences at larger percentiles. This is a desired property for summarizing which peptides have any magnitude for each of the properties, such as the net rule counts. Using either a linear scale or a logarithmic scale with a factor of one, would make small magnitudes difficult to distinguish from no magnitudes. In addition to the magnitude information this provides, we have included the property value sign information by moving the stack above the x-axis when the property is positive and to below the x-axis when the property is negative.

1. **Supplementary Figures and Tables**
   1. **Supplementary Figures**


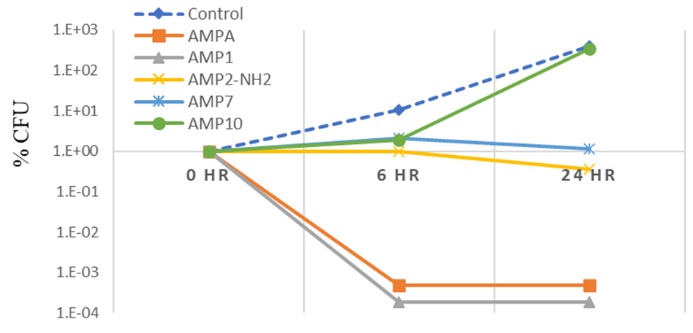


**Supplementary Figure S1a:** Antimicrobial activity against keystone periodontal pathogen *A. actinomycetemcomitans* for selected antimicrobial peptides. This data was used to supplement the training data of the CLN-MLEM2 model. AMPa: KWKLWKKIEKWGQGIGAVLKWLTTW-NH2, AMP1: LKLLKKLLKLLKKL, AMP2-NH2: KWKRWWWWR-NH2, AMP7: ESYKKML, AMP10: GILGKLWEGVKSTF


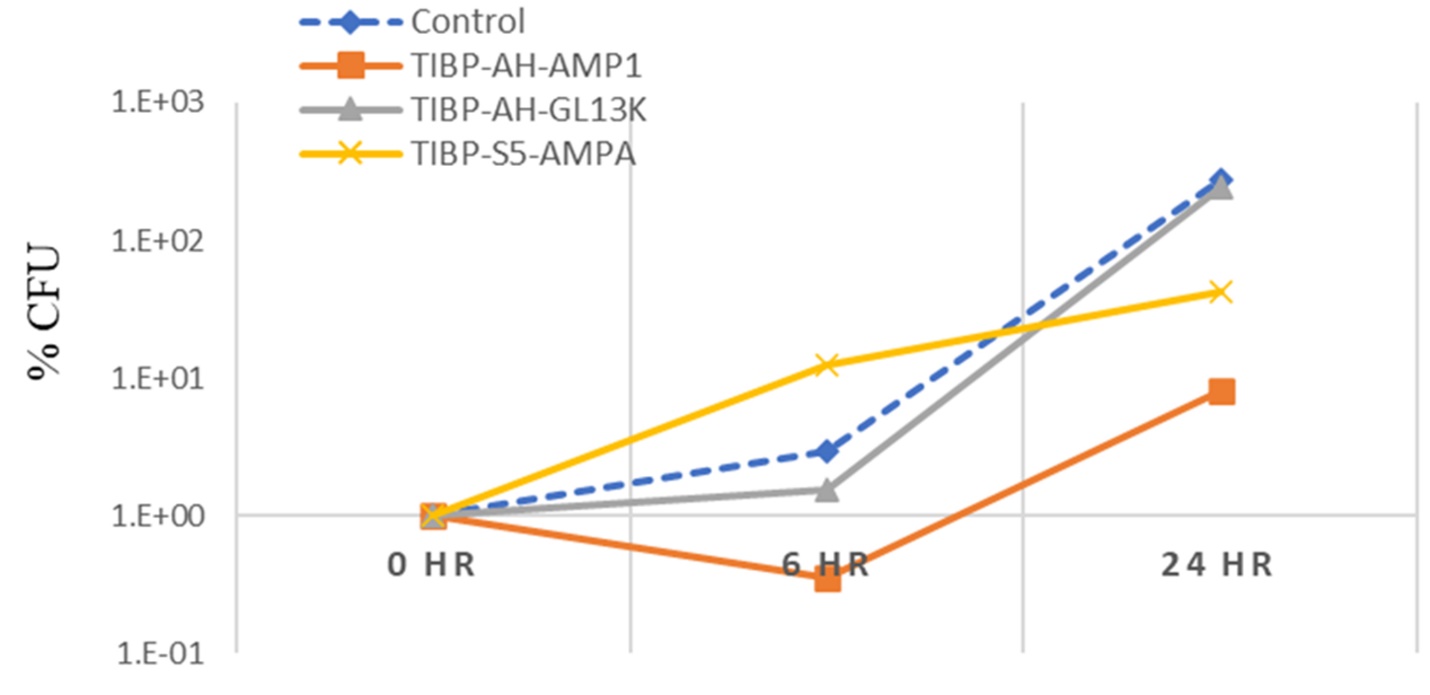


**Supplementary Figure S1b:** Antimicrobial activity against keystone periodontal pathogen *A. actinomycetemcomitans* for selected antimicrobial peptides. This data was used to supplement the training data of the CLN-MLEM2 model. TIBP-S5-AMPA: RPRENRGRERGLGSGGGKWKLWKKIEKWGQGIGAVLKWLTTW-NH2, TIBP-AH-AMP1: RPRENRGRERGLKGSVLSADLKLLKKLLKLLKKL, TiBP-AH-GL13K: RPRENRGRERGLKGSVLSADGKIIKLKASLKLL


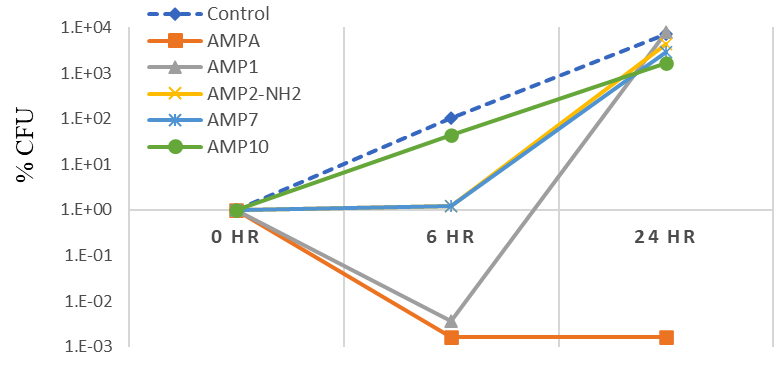


**Supplementary Figure S2a**: Antimicrobial activity against periodontal accessory pathogen *S. gordonii* for selected antimicrobial peptides. This data was used to supplement the training data of the CLN-MLEM2 model. AMPa: KWKLWKKIEKWGQGIGAVLKWLTTW-NH2, AMP1: LKLLKKLLKLLKKL, AMP2-NH2: KWKRWWWWR-NH2, AMP7: ESYKKML, AMP10: GILGKLWEGVKSTF


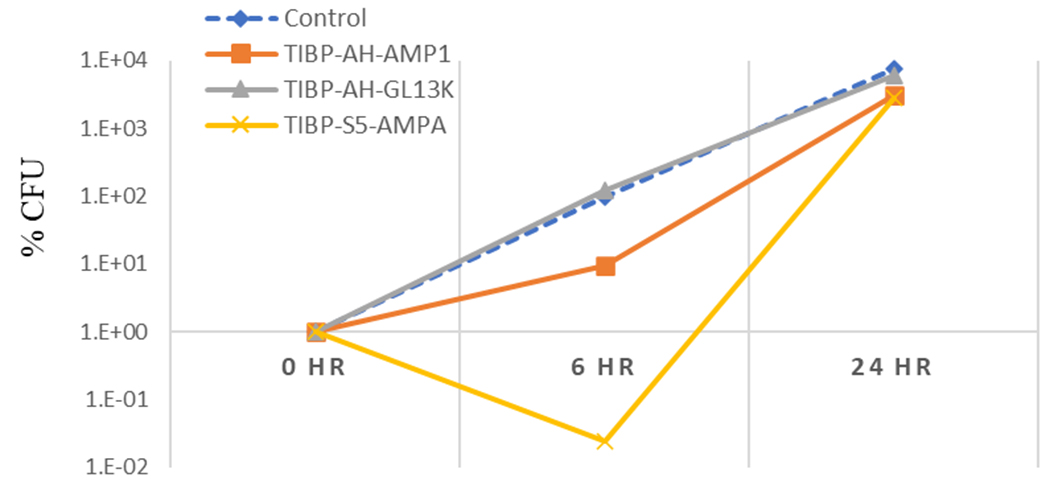


**Supplementary Figure S2b**: Antimicrobial activity against periodontal accessory pathogen *S. gordonii* for selected antimicrobial peptides. This data was used to supplement the training data of the CLN-MLEM2 model. TIBP-S5-AMPA: RPRENRGRERGLGSGGGKWKLWKKIEKWGQGIGAVLKWLTTW-NH2, TIBP-AH-AMP1: RPRENRGRERGLKGSVLSADLKLLKKLLKLLKKL, TiBP-AH-GL13K: RPRENRGRERGLKGSVLSADGKIIKLKASLKLL


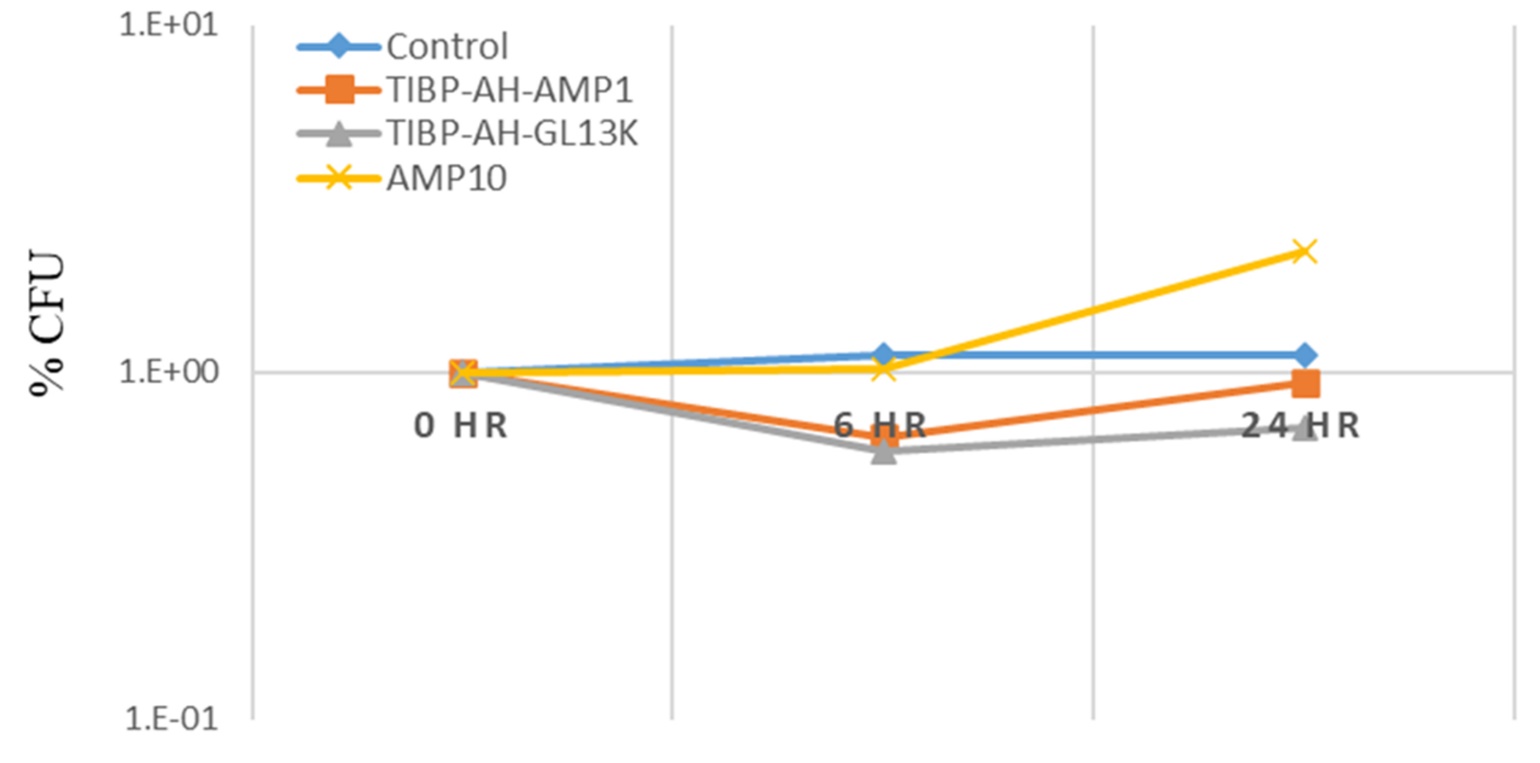


**Supplementary Figure S3:** Antimicrobial activity against periodontal accessory pathogen *P. gingivalis* for selected antimicrobial peptides. This data was used to supplement the training data of the CLN-MLEM2 model. TIBP-AH-AMP1: RPRENRGRERGLKGSVLSADLKLLKKLLKLLKKL, TiBP-AH-GL13K: RPRENRGRERGLKGSVLSADGKIIKLKASLKLL, AMP10: GILGKLWEGVKSTF


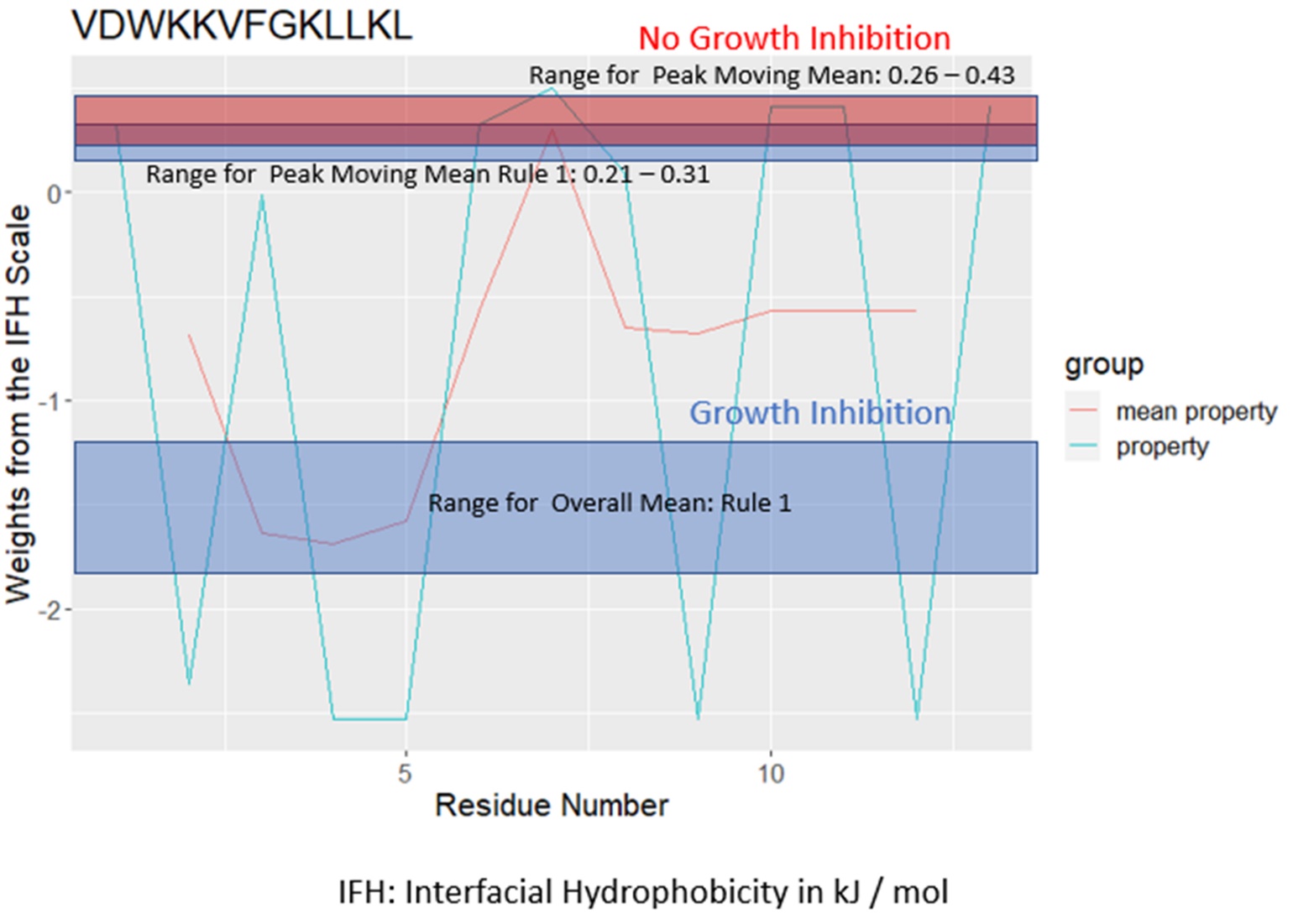


**Supplementary Figure S4:** Detailed Rough Set Theory Rule Evaluation for JACR890101 in the AAindex1 of VL-13. In Figure S10, the peak hydrophobicity features are compared for VL-13 and KF-18. While VL-13 met the first rule in Table S2, KF-18 did not. This plot shows the ranges of Rules 1 and 3 from Table S2, which correspond to *A. actinomycetemcomitans (Aa)* growth inhibition and no growth inhibition respectively. Rule 1 has two conditions for JACR890101: Condition 2 for peak hydrophobicity, indicating by a three amino acid (3-aa) hydrophobic feature, and Condition 4 for the mean hydropathy of the overall sequence to be hydrophilic. This description is a part of a quantitative boundary for amphiphilic sequence structure which is discriminating between inhibition and non-inhibition. Rule 3 Condition 4 also contains a hydrophobic 3-aa window. Rules are the conjunction of conditions, usually across multiple key physicochemical properties. Rule 1 (+ *Aa* inhibition) contains 5 conditions, Rule 2 (+ *Aa* inhibition) contains 4 conditions and Rule 3 (- *Aa* inhibition) contains 4 conditions.


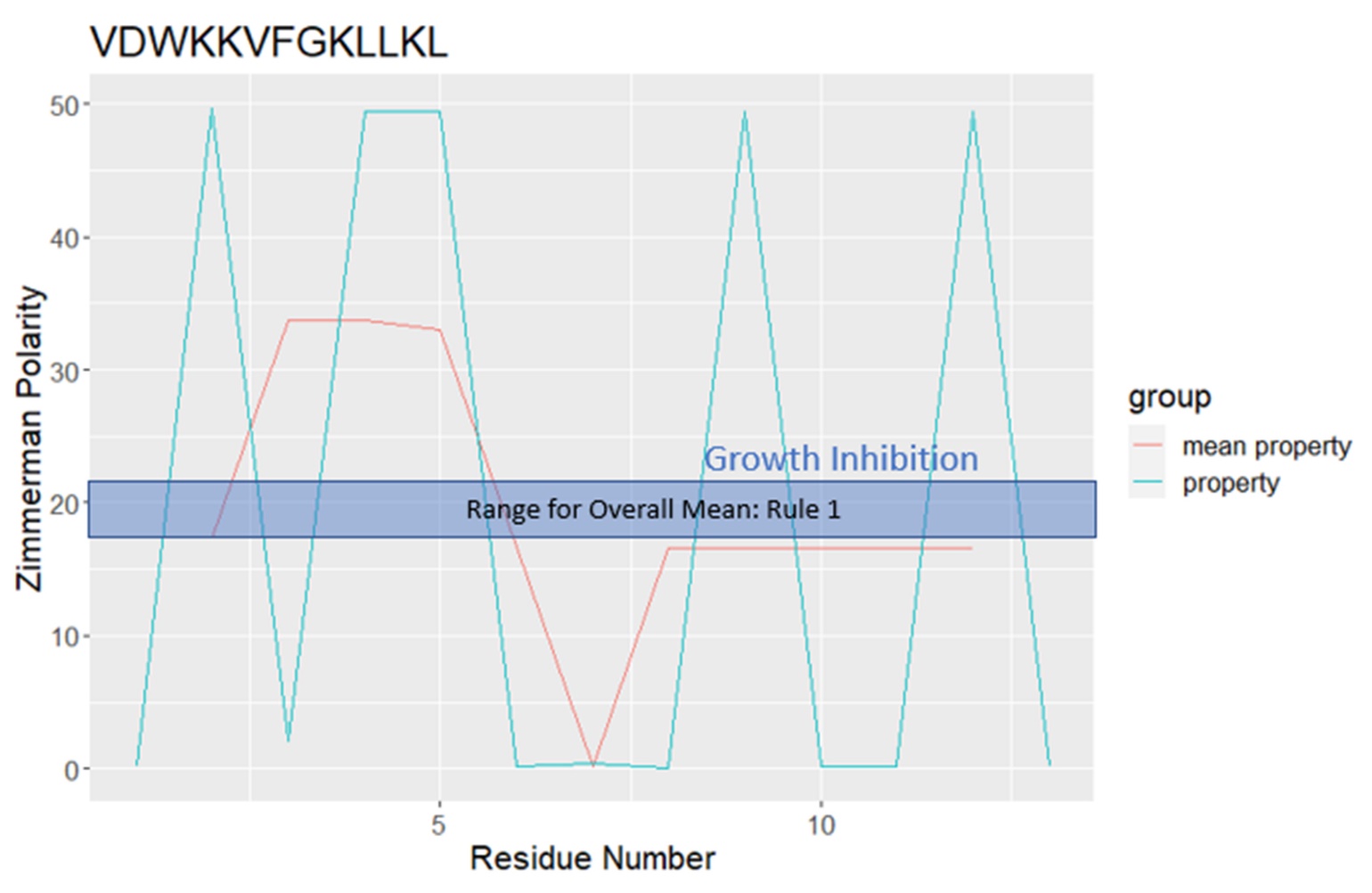


**Supplementary Figure S5:** Detailed Rough Set Theory Rule Evaluation for ZIMJ680103 in the AAindex1 of VL-13. VL-13 meets a total sequence characteristic of Rule 1 Condition 1 for this property. Rule 1 relates the overall mean of ZIMJ680103 (mean value: 13.6) to the probability of the amino acid components occurring in a sequence which is, on average, slightly hydrophilic (ranging from 17.1 to 23.0). Higher scores for this property are more hydrophilic. Rules are the conjunction of conditions, usually across multiple key physicochemical properties. Rule 1 (+ *Aa* inhibition) contains 5 conditions, Rule 2 (+ *Aa* inhibition) contains 4 conditions and Rule 3 (- *Aa* inhibition) contains 4 conditions.


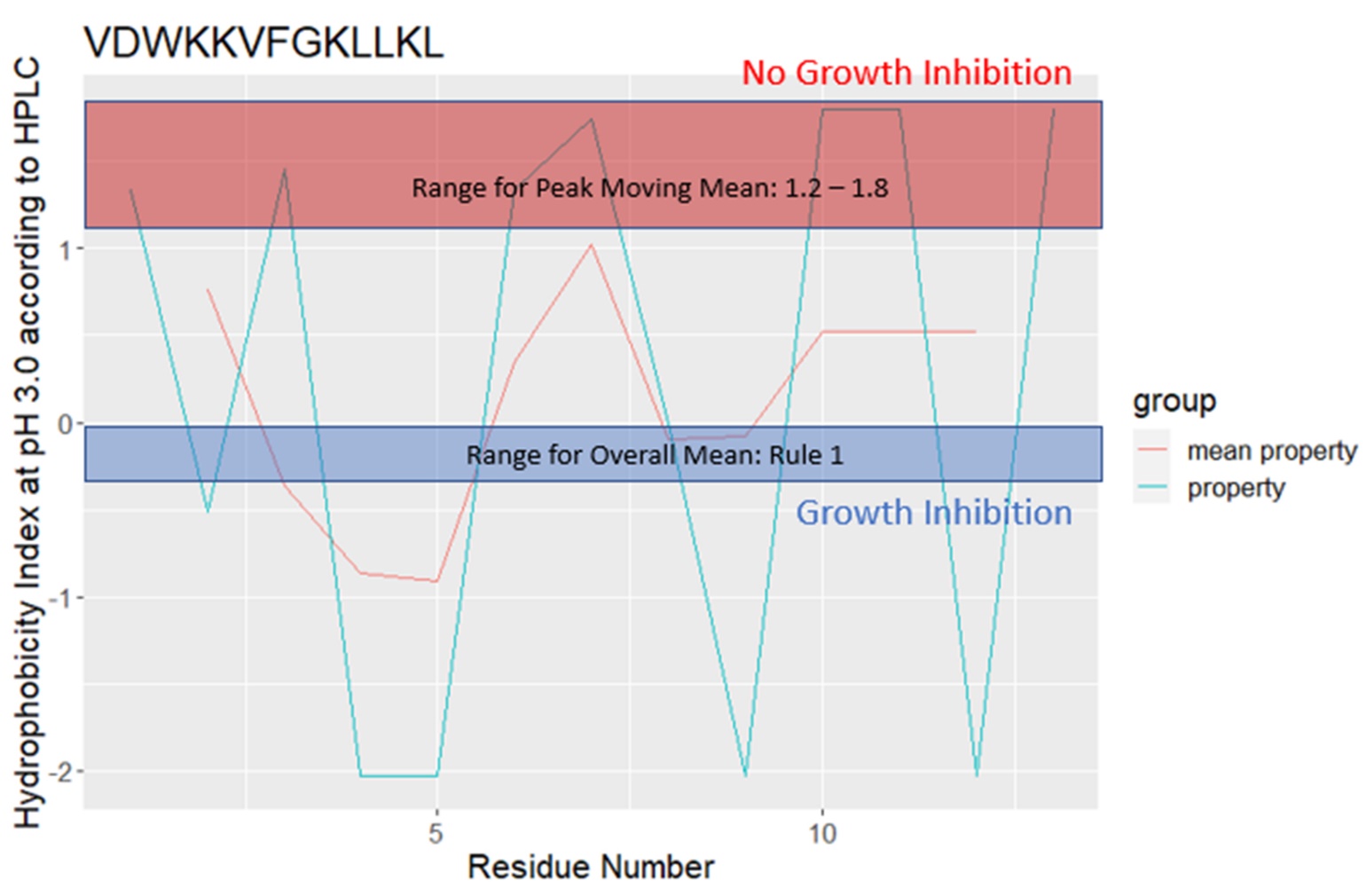


**Supplementary Figure S6:** Detailed Rough Set Theory Rule Evaluation for COWR900101 in the AAindex1 of VL-13. This property is contained in both Rule 1, in Condition 3 and in Condition 5, and Rule 3 Condition 2, partially discriminating between inhibiting the growth of *Aa* and its non-inhibition. This property relates to the changing polarity in acidic conditions. At pH 3, aspartic and glutamic acid side chain residues are uncharged and are counted as polar residues instead of charged residues. When sequences contain these two residues, this estimate will be less hydrophilic than for other hydropathy scales. For Rule 1 the sequence must average slightly hydrophilic average in acidic conditions as well have a sum of hydrophilic index between -27.82 and 0.27, indirectly constraining the length of peptides and hydropathy variation which meet Rule 1. Rule 3 requires hydrophobic peak under acidic conditions. Rules are the conjunction of conditions, usually across multiple key physicochemical properties. Rule 1 (+ *Aa* inhibition) contains 5 conditions, Rule 2 (+ *Aa* inhibition) contains 4 conditions and Rule 3 (- *Aa* inhibition) contains 4 conditions.


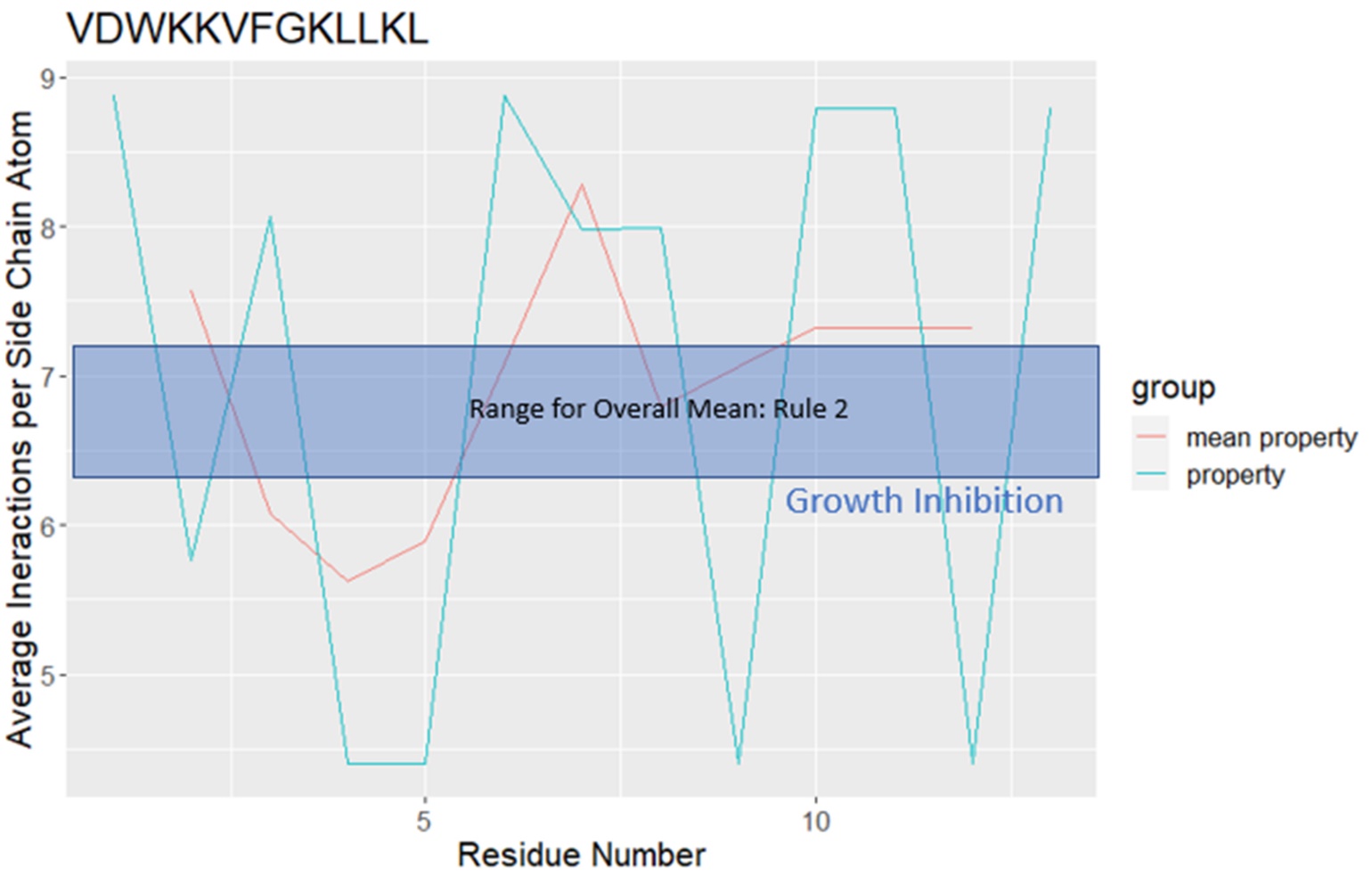


**Supplementary Figure S7:** Detailed Rough Set Theory Rule Evaluation for WARP780101 in the AAindex1 of VL-13. Peptides meeting Rule 2 Condition 1 include residues which average between 6.4 – 7.1 interactions per side chain, where the mean of the property is 7.4. Higher values tend to be less polar amino acids. Rule 2 contains a condition of being slightly non-polar for the total sequence. Therefore, VL-13 is slightly less polar by property WARP780101 and slightly polar by JACR890101 property because it also meets Rule 1 conditions. Being less polar and slightly polar is not a contradiction when different properties are considered. Rule 3 Condition 3 indicates that peptides in its set must as a highly non-polar 3-amino acid peptide window. Rules are the conjunction of conditions, usually across multiple key physicochemical properties. Rule 1 (+ *Aa* inhibition) contains 5 conditions, Rule 2 (+ *Aa* inhibition) contains 4 conditions and Rule 3 (- *Aa* inhibition) contains 4 conditions.


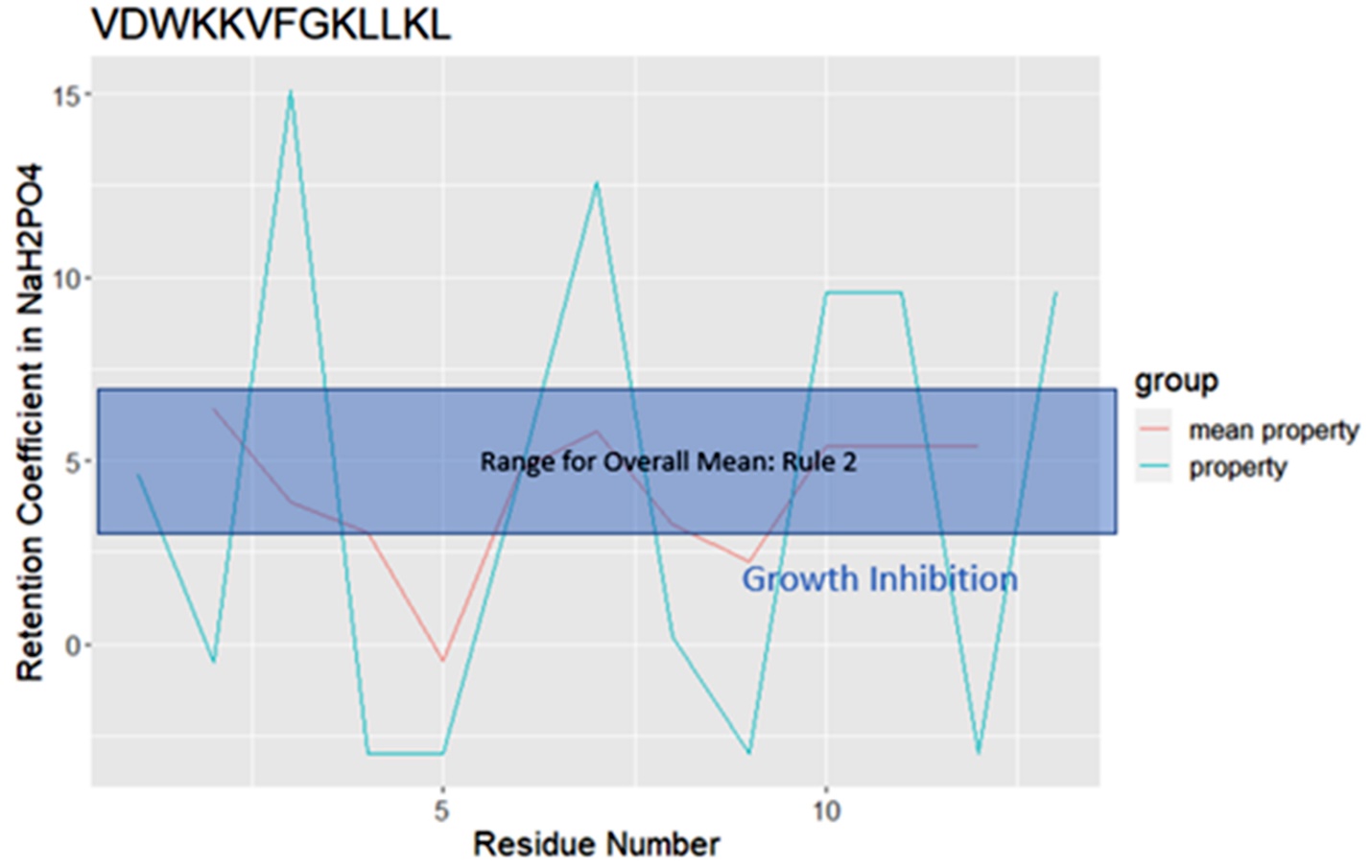


**Supplementary Figure S8:** Detailed Rough Set Theory Rule Evaluation for MEEJ810102 in the AAindex1 of VL-13. The key property is retention in NaH_2_PO_4_, with a pKa of 7.2. The mean of all of the amino acids is 2.6. The condition of Rule 2 Condition 2 for this property is an overall mean between 3.2 and 7.3, which is relatively hydrophobic for this hydropathy property. VL-13 is slightly overall non-polar by the Rule 2 description, but slightly overall polar by the Rule 1 description. Different polarity properties are used for the two rules. Rules are the conjunction of conditions, usually across multiple key physicochemical properties. Rule 1 (+ *Aa* inhibition) contains 5 conditions, Rule 2 (+ *Aa* inhibition) contains 4 conditions and Rule 3 (- *Aa* inhibition) contains 4 conditions.


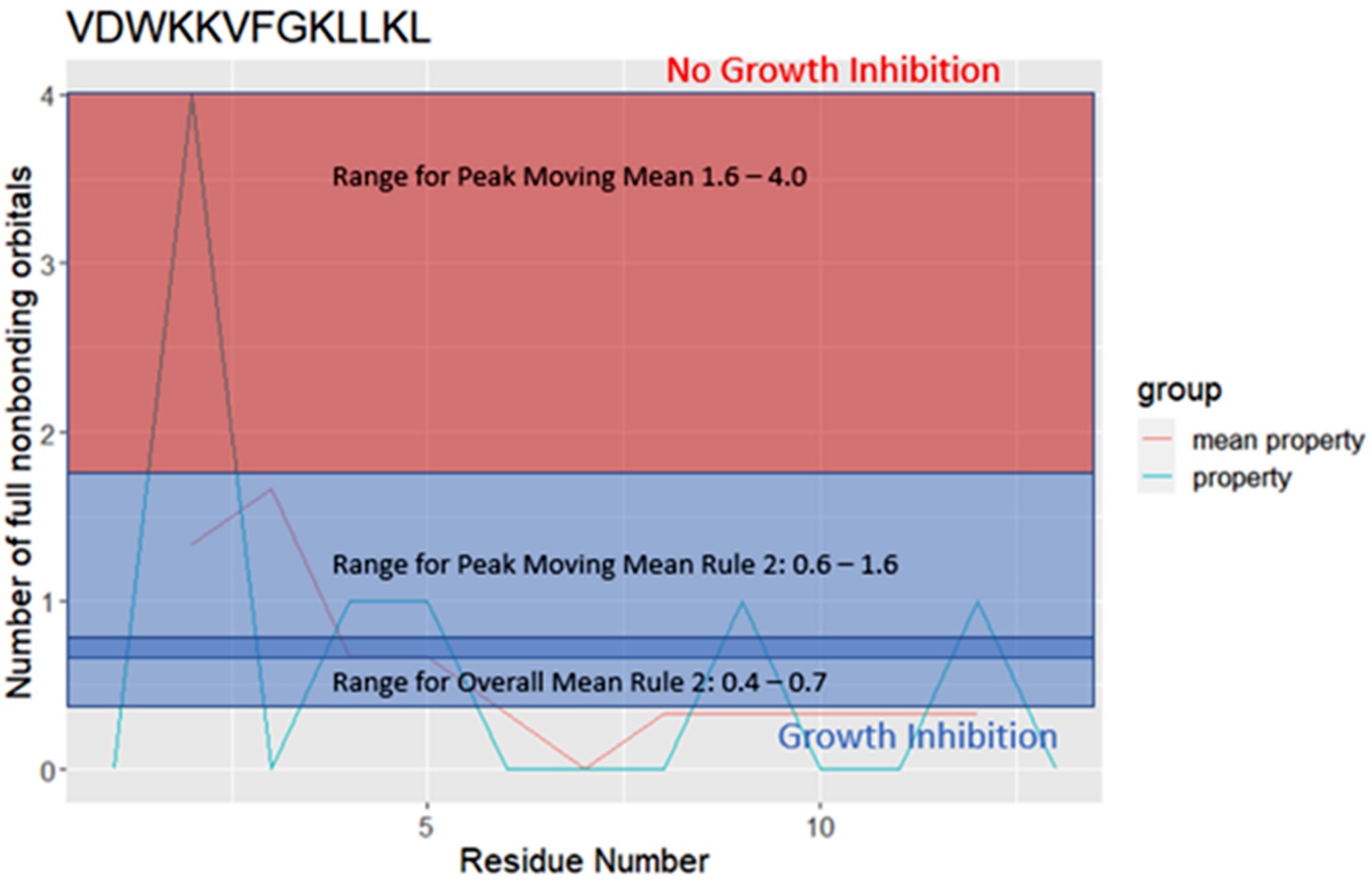


**Supplementary Figure S9.** Detailed Rough Set Theory Rule Evaluation for FAUJ880110 in the AAindex1 of VL-13. The number of full non-bonding orbitals is high for polar amino acids and tyrosine. Generally, hydrophobic amino acids have none. Having highly polar 3-aa window is Rule 3 Condition 1 that corresponds to non-growth inhibition. VL-13 does not meet this condition, but Rule 2 Condition 3 has a moderate polarity condition of a 3-aa feature for this property that VL-13 meets via residues 2-4 (DWK). A second condition for Rule 2 (Condition 4) is a moderately non-polar average between 0.4 and 0.69. The average for the property is 1.25 and lower values are less polar. Rules are the conjunction of conditions, usually across multiple key physicochemical properties. Rule 1 (+ *Aa* inhibition) contains 5 conditions, Rule 2 (+ *Aa* inhibition) contains 4 conditions and Rule 3 (- *Aa* inhibition) contains 4 conditions.

**Rough Set Theory Rule Summary for VL-13**

Both Rule 1 and Rule 2 have descriptions of amphipathy which are quantitative for targeting *Aa*. Using machine learning (ML) is important in this domain because a critical review of the features by CLN-MLEM2 has made distinctions which have hydropathy meaning in different formal senses, without losing technical rigor. ML can help make progress when the technical descriptions of properties change between these scales. Instead of being limited to selecting one scale to use, we use multiple scales with the traceability of knowing which scale is referenced at any given time. As an overview, targeting *Aa* seems to require amphipathic features, such as being generally hydrophilic, possessing one key hydrophobic 3-aa window, and also having a second 3-aa window which is moderately polar. For other properties, the rule is possessing a hydrophobic 3-aa window and being moderately non-polar. The lack of contradiction comes from which properties are chosen. The rule for a peptide not inhibiting *Aa* growth seems to be the possession of a 3-aa window of highly polar amino acids and a window which is hydrophobic by three different hydropathy descriptions.

Our machine learning model arranged the details of the known data, allowing for more precise predictions than discussed in the previous paragraph. This precision is not a safe guard against the discovered rules being inaccurate for future cases, but rather a guard against missing information due to vague, overlapping terminology. Because we have precise, conjunctive descriptions of when activity is predicted to occur, we benefit from automation for selection of sequences which meet these specific descriptions for activity. Our genetic algorithm makes finding sequences which meet multiple rules and conditions reliable.

- 1. **Supplementary Tables**

**Supplementary Table S1.** Antimicrobial peptides with known activity against periodontal pathogens. ‘NR’ indicates not reported (1). Pathogens are abbreviated as follows: *A. actinomycetemcomitans (Aa), S. gordonii (Sg), P. gingivalis (Pg).* MIC values higher than 100 μg/mL are not considered active for peptides. The MIC values for the proteins (nigrescinB and nigrescinC) are considered active below 100 mM. The activities of low and none are not distinguished in the targeting rule sets used for this study. Rules are distinguished between high activity and either low-activity or no-activity for each of the three bacterial species.

| *Sequence Name* | *Sequence* | *Aa Activity* | *Sg Activity* | *Pg Activity* | *Ref* |
| --- | --- | --- | --- | --- | --- |
| Pep7 | RPHGAGEGIDRVPAGPSPSEVGLAIPSGK | NR | NR | high | (2) |
| HBD3 | GIINTLQKYYCRVRGGRCAVLSCLPKEEQIGKCSTRGRKCCRRKK | high | NR | none | (3) |
| HBD2 | GIGDPVTCLKSGAICHPVFCPRRYKQIGTCGLPGTKCCKKP | none | NR | none | (3) |
| LL37/FALL39 | GSHMQVLSYKEAVLRAIDGINQRSSDANLYRLLDLDPRPTMDGDPDTPKPVSFTVKETVCPRTTQQSPEDCDFKKDGLVKRCMGTVTLNQARGSFDISCDKDNKRFA | none | NR | none | (4) |
| SMAP29 | RGLRRLGRKIAHGVKKYGPTVLRIIRIAG | high | NR | low | (4) |
| CAP18 | GLRKRLRKFRNKIKEKLKKIGQKIQGLLPKLA | high | NR | none | (4) |
| nigrescinC | MKTPSEFDDIRPFDSEELPEVYNRLLANEQFRQVLNYLYPNVPIDAISQKMHQCKTILEFQKAFAYTFLQELIDKASTGCDMESSAIDNTRQYTFVSNHRDIVLDSAFLSKLLIDNGFATTCEIAIGDNLLSLPWVRDLARLNKSFIVKRGLAPRELMQASVKMARYMIFALTEKHENLWIAQREGRAKDSNDLTQKSILKMFALGAEGTLLEKLQQFHVVPLSISYEYDPCDYLKAAEMQAKRDNANWKKGPMDDVLSMQTGIMGYKGNIHYHAAPCIDEYLETLKTTTDMANGPLLAAICQHIDKEIHRNYRLYANNYVALDELDGTTIYTNKYSDDNKAKFDAYIEKQLAKITLPNKDEAYLRQRLLEMYANPARNYLKANA | none | NR | high | (5) |
| nigrescinB | MKRTIAIVCGGDSSEHDVSLRSAQGLYSFFDKERYNIYIVDVKGTDWHVNLDNGHTAPINKNDFSFNNNGQTVFFDYAYITIHGRPGENGIMQGYFELVNIPYSTCDVLASALTFDKFVLNRYLKSYGIHVSESILLRLGETYNEREIAEKIGMPCFVKPATDGSSFGVSKVKNADQLAPALRKAFMESSEVMVESFLDGVEITQGIYKTREKSVPFPITEVVTSNEFFDYNAKYNGEVDEITPARISKELAEKVTEVTSHIYDILHANGIIRIDYIITKDNDGNDVINMLEVNTTPGMTVTSFVPQQVRAAGLDIKNVLSDIVENQF | none | NR | none | (5) |
| Adrenomedullin | YRQSMNNFQGLRSFGCRFGTCTVQKLAHQIYQFTDKDKDNVAPRSKISPQGY | NR | NR | high | (6) |
| GL13K | GLGKIIKLKASLKLL | NR | none | NR | (7) |
| KSL | KKVVFKVKFK | none | high | NR | (8) |
| Chrysophsin-1 | FFGWLIKGAIHAGKAIHGLIHRRRH | NR | high | NR | (9) |
| ZXR-2 | FKIGGFIKKLWRSLLA | NR | high | high | (10) |

**Supplementary Table S2.** Amino acid hydropathy properties selected in the CLN-MLEM2 rules for describing targeted antibacterial activity. These properties depict non-linear boundaries between activity types. *LIFS790102 (Conformational preference for parallel beta-strands) and PONP800108 (Average number of surrounding residues) were in referenced study, but not selected for rules.*

| ***AAindex1 ID*** | ***Reference Article Title*** | ***Scale Description*** | ***Reference*** |
| --- | --- | --- | --- |
|  |  |  |  |
| COWR900101 | “Hydrophobicity indices for amino acid residues as determined by high-performance liquid chromatography” | Hydrophobicity Index at pH 3.0 | (11) |
|  |  |  |  |
| FAUJ880110 | “Amino acid side chain parameters for correlation studies in biology and pharmacology” | Number of full non-bonding orbitals for each amino acid | (12) |
|  |  |  |  |
| JACR890101 | "The nature of the hydrophobic bonding of small peptides at the bilayer interface: implications for the insertion of transbilayer helices" | Interfacial Hydrophobicity Scale (IFH) | (13) |
|  |  |  |  |
| MEEJ810102 | “Prediction of peptide retention times in high-pressure liquid chromatography on the basis of amino acid composition” | Retention coefficients of amino acid residue at pH 2.1 | (14) |
|  |  |  |  |
| WARP780101 | A survey of amino acid side-chain interactions in 21 proteins | Evaluation of amino acid residues whose interaction frequencies differ by more than 50% versus expected | (15) |
|  |  |  |  |
| ZIMJ680103 | "The characterization of amino acid sequences in proteins by statistical methods" | Polarity scale for likelihood estimation | (16) |

**Supplementary Table S3.** Testing performance of the model targeting against *A. actinomycetemcomitans (Aa).* The rough set theory (RST) rules generated by CLN-MLEM2 model was tested on peptides reported as targeting oral bacteria and oral biofilms in a review by Sztukowska *et al*. (17). The match column describes if the sign of RST support, either positive or negative, matched the activity of the sequence. A “1” in “Match” indicates a match. A “0” indicates a mismatch. “0.5” was given for the case when the activity is dependent on an additional condition (Sequence #9: addition of an acetyl group at the C-terminal). RST Support is the number of training peptides which are identified by the rule set. False positive RST support is “FP”. If the rule is for activity, then the RST support is positive. If the rule is for inactivity, the RST support is negative. The weight is the magnitude of RST support. The area under the curve (AUC) weight is the product of the weight and the match values. Accuracy is the proportion of matches, and AUC is the ratio of the sum of the AUC weight column over the sum of the weight column. The AUC indicates that 76% of the RST support was classified correctly. Accuracy indicates 46% of test cases were classified correctly. FDR indicates 0% of weight relates to false positive predictions.

|  | Test Sequences | Match | False Positive | Aa RST Support | Weight | AUC Weight |
| --- | --- | --- | --- | --- | --- | --- |
| 1 | AKRHHGYKRKFH | 1 | 0 | -7 | 7 | 7 |
| 2 | ALWKTLLKKVLKAAA | 0 | 0 | -12 | 12 | 0 |
| 3 | ALWMTLLKKVLKAAAKAALNAVLVGANA | 1 | 0 | 16 | 16 | 16 |
| 4 | CAQAFGPNCVRLIVAVRIWRR | 1 | 0 | 16 | 16 | 16 |
| 5 | CMLPHHGACVRLIVAVRIWRR | 1 | 0 | 16 | 16 | 16 |
| 6 | DSHAKRHHGYKRKFHEKHHSHRGY | 1 | 0 | -6 | 6 | 6 |
| 7 | GLLWHLLHHLLH | 0 | 0 | 0 | 0.5 | 0 |
| 8 | IKKILSKIKKLLK | 1 | 0 | -2 | 2 | 2 |
| 9 | KRLFKKLLFSLRKY | 0.5 | 0 | 0 | 0.5 | 0.25 |
| 10 | RIWVIWRR | 0 | 0 | -7 | 7 | 0 |
| 11 | RRWIRVAVILRV | 0 | 0 | 0 | 0.5 | 0 |
| 12 | VRLIRAVRIWRR | 0 | 0 | 0 | 0.5 | 0 |
| 13 | VRLIVAVRIWRR | 0 | 0 | 0 | 0.5 | 0 |
| 14 | VRLIVRIWRR | 0 | 0 | 0 | 0.5 | 0 |
|  |  |  |  |  |  |  |
|  | Accuracy | 46% | FDR | 0.0% | AUC | 0.74 |

**Supplementary Table S4.** Testing performance of the model targeting against *P. gingivalis (Pg).* The rules generated by CLN-MLEM2 model was tested on peptides reported as targeting oral bacteria and oral biofilms in a review by Sztukowska *et al*. (17) The match column describes if the sign of RST support, either positive or negative, matched the activity of the sequence. A “1” in “Match” indicates a match. A “0” indicates a mismatch. “0.5” was given for the case when the activity is dependent on an additional condition (Sequence #9: addition of an acetyl group at the C-terminal). RST Support is the number of training peptides which are identified by the rule set. False positive RST support is “FP”. If the rule is for activity, then the RST support is positive. If the rule is for inactivity, the RST support is negative. The weight is the magnitude of RST support. The area under the curve (AUC) weight is the product of the weight and the match values. Accuracy is the proportion of matches, and AUC is the ratio of the sum of the AUC weight column over the sum of the weight column. The AUC indicates that 66% of the RST support was classified correctly. Accuracy indicates 68% of test cases were classified correctly. FDR indicates that 27% of weight relates to false positive predictions.

|  | Test Sequences | Match | FP | Pg RST Support | Weight | AUC  Weight |
| --- | --- | --- | --- | --- | --- | --- |
| 1 | AKRHHGYKRKFH | 0 | 16 | 16 | 16 | 0 |
| 2 | ALWKTLLKKVLKAAA | 0 | 21 | 21 | 21 | 0 |
| 3 | ALWMTLLKKVLKAAAKAALNAVLVGANA | 0 | 0 | -17 | 17 | 0 |
| 4 | CAQAFGPNCVRLIVAVRIWRR | 1 | 0 | 8 | 8 | 8 |
| 5 | CMLPHHGACVRLIVAVRIWRR | 1 | 0 | 8 | 8 | 8 |
| 6 | DSHAKRHHGYKRKFHEKHHSHRGY | 1 | 0 | 0 | 0.5 | 0.5 |
| 7 | GLLWHLLHHLLH | 1 | 0 | 25 | 25 | 25 |
| 8 | IKKILSKIKKLLK | 0 | 23 | 23 | 23 | 0 |
| 9 | KRLFKKLLFSLRKY | 0.5 | 7 | 14 | 14 | 7 |
| 10 | RIWVIWRR | 1 | 0 | 24 | 24 | 24 |
| 11 | RRWIRVAVILRV | 1 | 0 | 23 | 23 | 23 |
| 12 | VRLIRAVRIWRR | 1 | 0 | 23 | 23 | 23 |
| 13 | VRLIVAVRIWRR | 1 | 0 | 23 | 23 | 23 |
| 14 | VRLIVRIWRR | 1 | 0 | 23 | 23 | 23 |
|  |  |  |  |  |  |  |
|  | Accuracy | 68% | FDR | 27% | AUC | 0.66 |

**Supplementary Table S5.** Testing performance of the model targeting against *S. gordonii (Sg).* The rules generated by CLN-MLEM2 model was tested on peptides reported as targeting oral bacteria and oral biofilms in a review by Sztukowska *et al*. (17) The match column describes if the sign of RST support, either positive or negative, matched the activity of the sequence. A “1” in “Match” indicates a match. A “0” indicates a mismatch. “0.5” was given for the case when the activity is dependent on an additional condition (Sequence #9: addition of an acetyl group at the C-terminal). RST Support is the number of training peptides which are identified by the rule set. False positive RST support is “FP”. If the rule is for activity, then the RST support is positive. If the rule is for inactivity, the RST support is negative. The weight is the magnitude of RST support. The area under the curve (AUC) weight is the product of the weight and the match values. Accuracy is the proportion of matches, and AUC is the ratio of the sum of the AUC weight column over the sum of the weight column. The AUC of 0.49 indicates that 49% of the RST support was classified correctly. Accuracy of 39% indicates 39% of test cases were classified correctly. FDR indicates that 1.1% of weight relates to false positive predictions.

|  | Test Sequences | Match | FP | Sg RST Support | Weight | AUC  Weight |
| --- | --- | --- | --- | --- | --- | --- |
| 1 | AKRHHGYKRKFH | 1 | 0 | -36 | 36 | 36 |
| 2 | ALWKTLLKKVLKAAA | 1 | 0 | 20 | 20 | 20 |
| 3 | ALWMTLLKKVLKAAAKAALNAVLVGANA | 0 | 0 | 0 | 0.5 | 0 |
| 4 | CAQAFGPNCVRLIVAVRIWRR | 1 | 0 | 6 | 6 | 6 |
| 5 | CMLPHHGACVRLIVAVRIWRR | 1 | 0 | 53 | 53 | 53 |
| 6 | DSHAKRHHGYKRKFHEKHHSHRGY | 1 | 0 | -43 | 43 | 43 |
| 7 | GLLWHLLHHLLH | 0 | 0 | 0 | 0.5 | 0 |
| 8 | IKKILSKIKKLLK | 0 | 4 | 4 | 4 | 0 |
| 9 | KRLFKKLLFSLRKY | 0.5 | 0 | -25 | 25 | 12.5 |
| 10 | RIWVIWRR | 0 | 0 | -43 | 43 | 0 |
| 11 | RRWIRVAVILRV | 0 | 0 | -25 | 25 | 0 |
| 12 | VRLIRAVRIWRR | 0 | 0 | -25 | 25 | 0 |
| 13 | VRLIVAVRIWRR | 0 | 0 | -25 | 25 | 0 |
| 14 | VRLIVRIWRR | 0 | 0 | -43 | 43 | 0 |
|  |  |  |  |  |  |  |
|  | Accuracy | 39% | FDR | 1.1% | AUC | 0.49 |

**Supplementary References**

1. Grzymala-Busse JW. Mlem2—Discretization During Rule Induction. In: Kłopotek MA, Wierzchoń ST, Trojanowski, K, editors. *Intelligent Information Processing and Web Mining*. Berlin, Heidelberg: Springer (2003). p. 499-508. doi: 10.1007/978-3-540-36562-4_53.

2. Suwandecha T, Srichana T, Balekar N, Nakpheng T, Pangsomboon K. Novel Antimicrobial Peptide Specifically Active against Porphyromonas Gingivalis. *Archives of microbiology* (2015) 197(7):899-909. doi: https://doi.org/10.1007/s00203-015-1126-z.

3. Joly S, Maze C, McCray PB Jr, Guthmiller JM. Human Beta-Defensins 2 and 3 Demonstrate Strain-Selective Activity against Oral Microorganisms. *Journal of clinical microbiology* (2004) 42(3):1024-9. doi: https://doi.org/10.1128/jcm.42.3.1024-1029.2004.

4. Guthmiller JM, Vargas KG, Srikantha R, Schomberg LL, Weistroffer PL, McCray PB Jr, et al. Susceptibilities of Oral Bacteria and Yeast to Mammalian Cathelicidins. *Antimicrobial agents and chemotherapy* (2001) 45(11):3216-9. doi:  <https://doi.org/10.1128/aac.45.11.3216-3219.2001>.

5. Teanpaisan R, Narawatthana S, Utarabhand P. The Gene Coding for Nigrescin Produced by Prevotella Nigrescens Atcc 25261. *Letters in Applied Microbiology* (2009) 49(3):293-8. doi: https://doi.org/10.1111/j.1472-765X.2009.02657.x.

6. Dale BA, Fredericks LP. Antimicrobial Peptides in the Oral Environment: Expression and Function in Health and Disease. *Current issues in molecular biology* (2005) 7(2):119-34. https://doi.org/10.21775/cimb.007.119

7. Hirt H, Hall JW, Larson E, Gorr S-U. A D-Enantiomer of the Antimicrobial Peptide Gl13k Evades Antimicrobial Resistance in the Gram Positive Bacteria Enterococcus Faecalis and Streptococcus Gordonii. *PLOS ONE* (2018) 13(3):e0194900. doi: 10.1371/journal.pone.0194900.

8. Concannon SP, Crowe TD, Abercrombie JJ, Molina CM, Hou P, Sukumaran DK, et al. Susceptibility of Oral Bacteria to an Antimicrobial Decapeptide. *Journal of Medical Microbiology* (2003) 52(12):1083-93. doi: https://doi.org/10.1099/jmm.0.05286-0.

9. Wang W, Tao R, Tong Z, Ding Y, Kuang R, Zhai S, et al. Effect of a Novel Antimicrobial Peptide Chrysophsin-1 on Oral Pathogens and Streptococcus Mutans Biofilms. *Peptides* (2012) 33(2):212-9. doi: https://doi.org/10.1016/j.peptides.2012.01.006.

10. Chen L, Jia L, Zhang Q, Zhou X, Liu Z, Li B, et al. A Novel Antimicrobial Peptide against Dental-Caries-Associated Bacteria. *Anaerobe* (2017) 47:165-72. doi: https://doi.org/10.1016/j.anaerobe.2017.05.016.

11. Cowan R, Whittaker RG. Hydrophobicity Indices for Amino Acid Residues as Determined by High-Performance Liquid Chromatography. *Pept Res* (1990) 3(2):75-80. PMID: 2134053

12. Fauchere JL, Charton M, Kier LB, Verloop A, Pliska V. Amino Acid Side Chain Parameters for Correlation Studies in Biology and Pharmacology. *Int J Pept Protein Res* (1988) 32(4):269-78. doi: https://doi.org/10.1111/j.1399-3011.1988.tb01261.x

13. Jacobs RE, White SH. The Nature of the Hydrophobic Binding of Small Peptides at the Bilayer Interface: Implications for the Insertion of Transbilayer Helices. *Biochemistry* (1989) 28(8):3421-37. doi: 10.1021/bi00434a042.

14. Meek JL. Prediction of Peptide Retention Times in High-Pressure Liquid Chromatography on the Basis of Amino Acid Composition. *Proc Natl Acad Sci U S A* (1980) 77(3):1632-6. doi: 10.1073/pnas.77.3.1632.

15. Warme PK, Morgan RS. A Survey of Amino Acid Side-Chain Interactions in 21 Proteins. *J Mol Biol* (1978) 118(3):289-304. doi: 10.1016/0022-2836(78)90229-2.

16. Zimmerman JM, Eliezer N, Simha R. The Characterization of Amino Acid Sequences in Proteins by Statistical Methods. *J Theor Biol* (1968) 21(2):170-201. doi: https://doi.org/10.1016/0022-5193(68)90069-6.

17. Sztukowska MN, Roky M, Demuth DR. Peptide and Non-Peptide Mimetics as Potential Therapeutics Targeting Oral Bacteria and Oral Biofilms. *Mol Oral Microbiol* (2019) 34(5):169-82. Epub 20190815. doi: 10.1111/omi.12267.
